# Interdisciplinary framework for cyber-attacks and anomaly detection in industrial control systems using deep learning

**DOI:** 10.1038/s41598-025-89650-5

**Published:** 2025-07-22

**Authors:** Qawsar Gulzar, Khurram Mustafa

**Affiliations:** Department of Computer Science, Jamia Millia Islamia, Okhla, New Delhi, 110025 India

**Keywords:** Industrial control systems (ICS), Intrusion detection system (IDS), Machine learning (ML), Deep learning (DL), Mutual information (MI), Sparse principal component analysis (SPCA), Energy science and technology, Engineering, Materials science

## Abstract

The reliability of Industrial Control Systems (ICS) is crucial given their substantial importance in society and business. These systems are susceptible to physical and cyber-attacks, which can seriously affect human life and the economy. Given the growing prevalence of Internet of Things (IoT) technologies and the possibility of cyber warfare, it is essential to prioritize safeguarding Industrial Control Systems (ICS) from cyberattacks. Previous studies revealed an imbalance in the ICS datasets. As a result, models didn’t do well in minority classes but did well in majority classes, which made the intrusion detection system (IDS) less useful. The key objective is to provide insights into the normal functioning of the system and the disruptions produced by cyberattacks. In this study, we introduced an interdisciplinary framework that aims to enhance network intrusion detection systems (NIDSs). In this framework, we introduced an IDS via feature selection and feature reduction technique(s) with the attention-driven lightweight deep neural networks: Deep Recurrent Neural Networks (RNN), Deep Long Short-Term Memory (LSTM), and Deep Bi-directional Long Short-Term Memory (Bi-LSTM). Several feature selection techniques exclude features that fail to match the specified criteria. We employed Sparse Principal Component Analysis (SPCA) to extract higher-order features. We conducted experiments on the three datasets: the Secure Water Treatment System (SWaT), the Water Distribution (WADI), and the Gas Heating Loop (GHL). Among the models used in the framework, the attention-driven Deep LSTM model produced better results than the others, revealing lower training and testing times for the three datasets. In terms of precision, recall, F1-score, computational speed, and ability to work with larger datasets and different ICSs, the proposed framework is better than previous methods in detecting cyberattacks. This highlights how useful it is in the real world.

## Introduction

Industrial control systems (ICSs), or Supervisory Control and Data Acquisition (SCADA) systems, combine distributed processing elements with tracking and governing the physical process. These systems include components that collect feedback from the physical environment (sensors) and components that affect the environment (actuators). The feedback data is processed by the computers and controller networks, which then generate instructions for the actuators. Many Industrial Control Systems (ICS) are critical in terms of safety, where any disruption to their operational integrity can result in significant financial and environmental repercussions and jeopardise the well-being of individuals and national security. The proliferation of industrial Internet of Things (IIoT) technology has increased possible attack spots and vulnerabilities inside the system.

The significance of ICS makes them appealing targets for possible attacks. In recent years, there have been several notable events with major repercussions. These include the attacks on power infrastructure in Ukraine^1^, the well-known Stuxnet malware that specifically targeted nuclear centrifuges in Iran^2^ and the recurrent assaults on the Saudi oil business^3^. The frequency of ransomware attacks against ICS, with the objective of financial gain, is rising^4^. In the first six months of 2021, “DarkSide, a Russian hacker, claimed responsibility for a ransomware attack on the US fuel pipeline Colonial Pipeline,” causing the company to disable SCADA systems and remit $5 million in cryptocurrency^5^. Cybersecurity in Industrial Control Systems (ICS) has seen significant advancements in dealing with the constraints and vulnerabilities present in the existing system for detecting cyber-attacks in industrial settings.

Cyber-attack detection systems are intended to track activities inside an information system and spot indicators of potential security breaches. The technique most often used for detecting attacks is anomaly detection, which involves identifying events that deviate from the anticipated behaviour of the system. One of the primary inherent benefits of using the anomaly detection technique is its capacity to identify previously unnoticed and novel attacks. A multitude of Machine Learning (ML) methods^6^ from supervised learning such as Support Vector Machine (SVM)^7,8^, Principal Component Analysis (PCA)^9^, Neural Networks^10^, from unsupervised learning such as Cluster Analysis^11^, Negative Selection Algorithm (NSA)^12^ and so on, from reinforcement learning such as Q-learning, SARSA, Deep Q-Network (DQN) and others may be used to build attack detection strategies based on anomaly detection. Conventional machine learning techniques are designed to detect cyber-attacks on ICS that have the potential to cause system malfunctions and disrupt the regular operation of physical processes. However, misclassifying highly detrimental traffic flows frequently arises from inaccurate and insufficient feature selection in specific machine learning models. Nevertheless, the accurate identification of cyber-attacks on ICS continues to pose a significant issue in cases when the systems are exceedingly complicated, and there exists a scarcity of information about the subject under investigation. Deep neural networks^13–16^ have shown considerable efficacy in addressing the challenge of adaptability in the context of cyber-attack detection on industrial control systems (ICSs).

Deep Learning is a cutting-edge approach of Artificial Intelligence (AI), including methods and solutions that greatly enhance the potential and efficacy of neural networks^17,18^. The neural network may create a chosen object with many layers by starting with basic characteristics and working on more complicated ones.

Despite several effective approaches for diagnosing device issues caused by cyber-attacks, it has been shown that deep learning-based solutions do not consistently exhibit stability in their performance^19^. The method’s limits depend on how complicated the physical system is. We aim to get beyond this restriction and create an interdisciplinary framework. This approach compares the benefits and detriments inherent in distinct neural networks with each other. It will guarantee minimal correlation of errors among the models employed in the framework and high precision in detecting cyber-attacks.

The widespread use of IoT devices often leads to a significant increase in network traffic. As a result, differentiating between a regular rise in traffic and an unexpected surge, which includes cyberattacks, may be tough. The detection of adverse attacks requires information to address these issues successfully. The methodology suggested in our research assumes a sophisticated adversary that aims to alter the physical-level process of the targeted ICS. The adversary in consideration has successfully infiltrated the system and can manipulate sensor data, issue harmful directives to the actuators, and deceive system operators by altering the network traffic between the field process and the control centre.

The primary objective of this framework is to optimize the deep learning techniques to evaluate the effectiveness of our models in detecting complex cyberattacks, including Single-Stage Single-Point (SSSP), Single-Stage Multi-Point (SSMP), Multi-Stage Single-Point (MSSP), and Multi-Stage Multi-Point (MSMP) attacks, in the SWaT testbed. The cyberattack modalities in the context of the WADI testbed. Unauthorized modifications to the GHL dataset may include changes to the maximum RT level, maximum HT temperature, pump frequency, and system relaxing time value. The intention was to carry out these assaults on the secure water treatment system (SWaT) testbed, which includes sensors, actuators, and Programmable Logic Controllers (PLC). The purpose is to proactively prevent such cyber-attacks in real-world Industrial Control Systems (ICS). The main contributions of our study are as follows:

1. After evaluating various feature selection strategies, we found that mutual information (MI) significantly improved the model’s performance in cyberattack detection by selecting the best feature subsets. A cross-validation method was used to establish the ideal feature significance threshold.
2. To deal with larger-dimensional data, it is possible to create sparse eigenvectors by using Sparse Principal Component Analysis (SPCA) for feature reduction, which forces a collection of lesser-value loads to be zero.
3. An IDS based on the integration of feature selection and feature reduction techniques with the attention-driven lightweight deep neural networks: Deep Recurrent Neural Networks (RNN), Deep Long Short-Term Memory (LSTM), and Deep Bi-directional Long Short-Term Memory (Bi-LSTM), is used to record insights into how a system operates in the normal mode and the disruptions brought on by cyberattacks.
4. Complex datasets with large data volumes and a multitude of features that replicated actual ICS scenarios were used to test the suggested IDS models.

The subsequent sections of the paper are structured in the following manner. Section “Related works” overviews the literature on ICS cyberattacks and anomaly detection. This includes an examination of several methodologies, such as machine learning and deep learning, that have been used in this domain. Section “Methodology” introduces an interdisciplinary framework consisting of three attention-enabled deep learning models that use varied feature selection techniques for feature selection. Additionally, the framework incorporates sparse PCA to reduce the dimensionality of the features to retain the higher-order features. The description of the experimental datasets, the evaluation metrics used, and the presentation of the experimental findings are in Section “Experimental results”. Section “Discussion” discusses the pros and cons of the proposed framework. Lastly, the concluding remarks and some future insights are incorporated.

## Related works

Several methods have been put forward to identify cyberattacks in Industrial Control Systems (ICSs). Several examples include the use of rule-based Intrusion Detection Systems (IDS)^20,21^ or Deterministic Finite Automata (DFA)^22^. However, at the moment, a majority of the works are concentrated on creating new methods or expanding on current ones by using cutting-edge technologies like Big Data^23^ or ML/DL. Specifically, there has been a growth in the use of ML and DL methods, which serve an important role in spotting cyber-attacks in the industrial sector^24^. In this part, we do an in-depth evaluation of the best research on ML/DL for anomaly detection in the industry sector. The most important points of these works are summarized in Table 1.

**Table 1:** Summary of numerous works that focused on anomaly detection in ICS settings.

References	Dataset	Data pre-processing	Feature selection	Feature extraction	Anomaly detection method	Year
^25^	SWaT	Selecting features using testing	NA	NA	DNN	2017
^26^	SWaT	Signal denoising, separation, and synchronization	NA	Partial	Bayesian Group	2018
^19^	SWaT	Scaling features and the handling of corrupted values	NA	Current-past feature value difference	CNN and LSTM	2018
^27^	–	Feature scaling and one-hot encoding	Analyze the statistical distribution	NA	LSTM	2019
^28^	SWaT	Feature scaling	NA	NA	LSTM	2019
^29^	SWaT, WADI, and BATADAL	Feature scaling	Kolmogorov–Smirnov test	Fourier	1DCNN AE	2019
^6^	SWaT, and, WADI	Feature scaling	NA	PCA	DIF	2020
^30^	SWaT	Feature scaling, one-hot encoding, and treatment of missing values	Correlation filter, Kolmogorov–Smirnov test, variance test	Fourier and autocorrelation	LSTM	2020
^16^	SWAT, and GHL	Feature scaling	NA	NA	1D-CNN, LSTM, and GRU	2021
^31^	CWSS	Denoising, feature scaling	Chi-square, and correlation-based	NA	SVM, k-NN, MLP, DT, and RF	2021
^32^	Power System dataset and UNSW-NB15 dataset	Gaussian mixture model	Kalman Filtering	NA	CNN	2022
^33^	Gas Pipeline and UNSW-NB15 dataset	NA	NA	Slave MODBUS in the given window time	Autoencoder, LSTM	2022
^34^	Gas Pipeline	Feature Selection, Normalization	NA	ICA	Federated-SRU	2023
^35^	MNIST, KDDCup, Amazon	Feature Selection, Normalization	Mahalanobis Distance	Gaussian Mixture Models	PPFL	2024
^36^	BoT-IoT, UNSW-NB15,and N-BaIoT	Feature scaling and one hot encoding	Modified Genetic Algorithm	NA	LSTM	2024
^37^	SWaT, and WADI	NA	Genetic Algorithm	NA	LSTM	2025
Ours	SWaT, WADI, and GHL	Feature scaling, and treatment of missing or corrupted values, Normalization	Mutual Information, Information Gain, Correlation Based	SPCA	RNN, LSTM, and Bi-LSTM	–

Every column reflects the actions we thought would best detect cyberattacks in the situations involved.

Anomaly detection in ICSs is often seen as a time-series classification challenge because of its intrinsic nature. As a result, many approaches depend on time-efficient algorithms.

Besides CNN and LSTM networks, other Deep Learning (DL) and Machine Learning (ML) methods have been proposed and discussed in the literature. In Inoue et al.^25^, authors proposed and evaluated unsupervised machine learning algorithms for identifying ICS irregularities. This study compared the Deep Neural Network (DNN) with the Support Vector Machine (SVM). Both approaches now work with time-series data. Use of mean and standard deviation normalized validation dataset. The method completely lacks the prediction information for detecting the cyberattacks.

Using an approach based on graphical models, the operational ICS called SWaT is utilized to assess expected operational behaviour^26^. The Bayesian Network is used to train timed automatons to discover correlations between sensors and actuators. The precision of the graphical models enables the analysis of detection results and the identification of malfunctioning sensors or actuators. The model attained high false positives.

Due to its nature, anomaly detection in information-rich systems is generally treated as a time-series classification problem. Thus, several methods use temporal algorithms. CNN and LSTM neural networks are noteworthy deep learning algorithms. Both methods can handle temporal aspects and turn incoming data into sequential representations. In one study^19^, researchers tested several deep-learning models for anomaly detection. We tested the Long Short-Term Memory (LSTM) and 1D-Convolutional Neural Network (1D-CNN) methods. The authors also suggested adding new characteristics by calculating the difference between present and preceding values using a lag. The researchers found that the One-Dimensional Convolutional Neural Network (1D-CNN) outperformed the long short-term memory (LSTM) model and trained faster. The method is restricted to SWaT dataset only.

An IDS built on the LSTM neural network was introduced^27^. The researchers put out a Cumulative Sum (CUSUM) technique to detect anomalies. The Cumulative Sum (CUSUM) is derived by evaluating the residual error obtained by comparing the ground-truth data with the model’s anticipated data. The proposed IDS underwent into delayed attack detection.

The authors in^28^ suggested a sequence-to-sequence LSTM neural network for outlier detection, which is another use of LSTM networks. In its training, the LSTM network used an attention mechanism and was fed with data collected under normal settings.

Using ID-CNN and autoencoders, a unique method was developed to detect attacks. The effectiveness of this method was assessed using basic, resource-efficient neural networks on three open datasets^29^. This approach can survive deliberate attacks that exploit the neural network’s vulnerabilities, allowing adversaries to accomplish their physical outcomes without notice. Although the method achieved good results on one end but increased the computational complexity on the other side. In their study, the researchers presented an anomaly detection Intrusion Detection System (IDS). They constructed the IDS using ML techniques, including Naive Bayes, PART, and Random Forest (RF). Additionally, they conducted a feature extraction procedure to enhance the system’s performance.

The authors of^6^ proposed a semi-supervised learning method. This method trains two Isolation Forests (IF). One IF uses normalized copies of the original data, while the other uses variants of similar datasets pre-processed using Principal Component Analysis (PCA). The demerits of the proposed method is a limited number of attacks were detected and lack applicability to higher dimensional datasets.

Prior research^30^ introduced “MADICS” as a revolutionary anomaly identification method. Deep learning was applied to mimic Industrial Control System behaviour. Thus, they addressed a gap in machine learning and deep learning model construction methods for ICS cyberattack detection. The proposed methodology predicted 23 out of 36 attacks and requires a semi-supervised prepared SWaT dataset.

The Hybrid DeepGCL model incorporates the SPOCU activation function^16^ into CNN, GRU, and LSTM hidden layers. This integration aids interdisciplinary ICS cyberattack detection. These ICSs provide both benign and malignant system inputs. The proposed model is limited to 15 epochs due to the computational overhead of merging the models.

The authors in^31^ suggest implementing an energy monitoring system for actuators and sensors in Industrial Control Systems (ICSs). This monitoring system measures voltage, current, and power via a hard-wired INA219 current sensor. Using this strategy for anomaly detection across many ML algorithms, including Support Vector Machines (SVM), k-nearest Neighbors (k-NN), Multilayer Perceptron (MLP), Decision Trees (DT), and Random Forests (RF). The researchers selected features using information gain, chi-square, and correlation. The proposed system requires improvement in detecting multisource coordinated attacks.

The authors in^32^ developed a technique for identifying anomalies by integrating a deep learning technology, Convolutional Neural Network (CNN), with a Gaussian-Mixture Model (GMM) based on Kalman Filter (KF). The proposed framework has two key steps. Pre-process and filter data to ensure security. Their GMM-KF deep CNN anomaly detection model accurately evaluated the posterior probability of abnormal and genuine ICSs.

The authors presented a deep autoencoder-based Intrusion Detection System (IDS) to accurately differentiate between malicious operations and legitimate activities in Industrial Internet of Things (IIoT) driven Industrial Control Systems (IICS) networks in real-time. The proposed model utilizes a Long Short-Term Memory (LSTM) autoencoder architecture to accurately detect intrusive events inside the IICS networks^33^. How to secure such systems against advanced cyberattacks is missing.

The authors introduced a novel Intrusion Detection System (IDS) paradigm called federated-simple recurrent units (SRUs) for safeguarding Internet of Things (IoT) based Industrial Control Systems (ICSs). The federated-SRUs IDS model employs an enhanced design of simple recurrent units to decrease computing expenses and address the problem of gradient vanishing in recurrent networks. Next, it conducts data aggregation utilizing many communication rounds inside the federated architecture, enabling various ICS networks and stakeholders to collaboratively construct a comprehensive IDS model while ensuring the privacy of the data^34^. The task involves optimizing the weights for communication between local and global servers in the IDS model using asynchronous federated learning approaches. Additionally, it needs testing the model on numerous datasets.

The authors in^36^ presents an innovative use of the Modified Genetic Algorithm (MGA) for feature selection and the Genetic Algorithm (GA) for optimizing LSTM parameters inside an Edge Computing framework for cyberattack detection. The research, however, executed proficiently, necessitates application on varied datasets, with an increase in epoch count.

The authors in^37^ presented a novel intrusion detection model for the Industrial Internet of Things (IIoT) utilizing a genetic algorithm with an attention mechanism and a modified Adam-optimized Long Short-Term Memory (GA-mADAM-IIoT) for detecting intrusion threats in IIoT environments. Future research will focus on integrating privacy preservation and blockchain technology to create a threat detection system particularly designed for industrial networks.

In response to these issues, the researchers have developed a resilient privacy-preserving federated learning model that can withstand model poisoning attempts while maintaining accuracy. Their method involves the use of an internal auditor to assess the homogeneity and dispersion of encrypted gradients. This allows us to distinguish between harmless and harmful gradients. We apply a Gaussian Mixture Model and Mahalanobis Distance to ensure our aggregation process is resilient to malicious attacks. The suggested approach employs Additive Homomorphic Encryption to provide secrecy while minimizing computational and transmission cost^35^. Despite producing positive results, the model still has to be strengthened to withstand the numerous threats that edge computing, blockchain, and quantum computing pose.

In summary, most of the researchers have introduced machine learning and deep learning-based IDS in securing ICS. Intermediate steps are needed to improve Machine Learning (ML) and Deep Learning (DL) models for identifying cyber-attacks in ICS settings. The performance of deep learning neural networks on large-dimensional datasets has not been compared. This research presents a general-purpose, deep-learning-based NIDS framework that may be used for various balanced data types. It checks separately the efficacy of an attention-driven Deep Recurrent Neural Network (RNN), Deep Long Short-Term Memory (LSTM) neural network, and a Deep Bi-directional Long Short-Term Memory Neural Network (Bi-LSTM) for identifying cyberattacks that cause ICS failures. The proposed framework harnesses the benefits of the best feature selection technique from the multitude of feature selection groupings and the feature reduction technique to extract the higher-order features for better model convergence. The proposed framework is validated by applying it to three different real-time datasets: the Secure Water Treatment Dataset (SWaT)^38,39^, the Water Distribution (WADI) dataset^40^, and the Gasoil Heating Loop (GHL) dataset^41,42^ which includes details regarding the typical behaviour of ICS and the breaches brought on by cyberattacks. Moreover, its implementation outperforms popular machine learning and deep learning techniques in identifying ICS cyberattacks, such as simple deep neural network (DNN)^25^, support vector machines (SVM)^25^, Time Automata and Bayesian network (TABOR)^26^, Long Short-Term Memory (LSTM) neural networks^28^, DeepGCL^16^, and Convolutional Neural Network (CNN)^19^. Furthermore, the catch is that deep neural networks’ individual and all-encompassing powers are not considered. So, the relevance of our suggested study is justified.

## Methodology

**Fig. 1:**
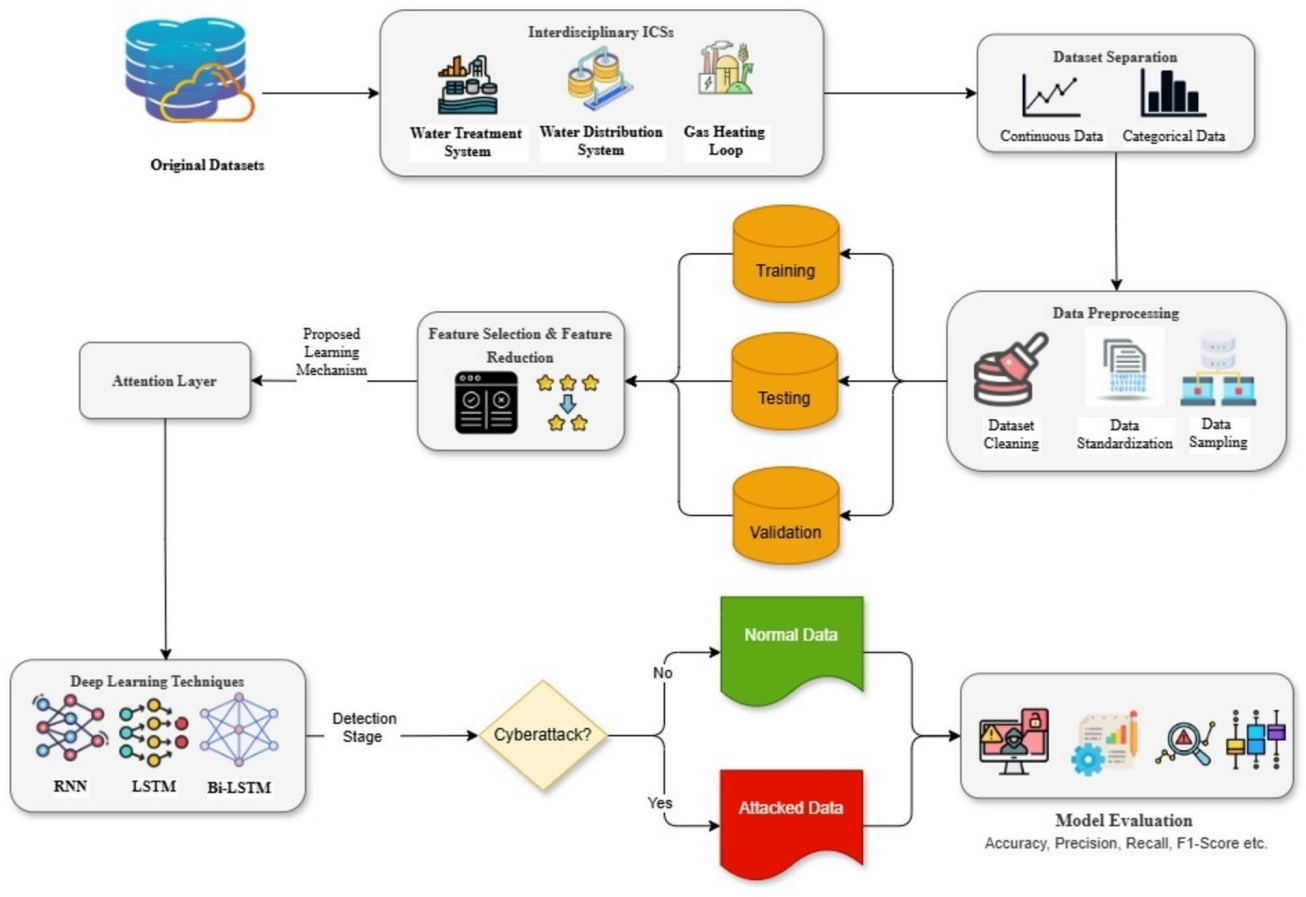
Methodology of the proposed architectural framework.

The proposed framework is constructed by leveraging the normal process data and the malignant process data of the sensor measurements. This framework introduces an IDS via feature selection and feature reduction with attention-driven lightweight deep neural networks: Deep Recurrent Neural Networks (RNN), Deep Long Short-Term Memory (LSTM), and Deep Bi-directional Long Short-Term Memory (Bi-LSTM). These models are especially well-suited for anomaly detection in Industrial Control Systems (ICS) because they can capture temporal relationships in sequential data, which is an important property of ICS network traffic. The task of cyberattack detection by the proposed framework accompanied by the necessary steps, is shown in Fig. 1 below.

### Dataset preprocessing unit

Machine Learning (ML) uses algorithms and statistical models to analyze data and construct predictive models that predict outcomes. Machine learning models depend heavily on their development data. The details and descriptions of data affect the efficacy of the machine learning model. When presented with difficult, repetitious, or noisy raw data, the model may struggle to gain useful insights for the intended application. It comprises data cleaning, feature extraction, dimensionality reduction, noise filtering, etc. Many data pre-processing methods are employed. Technical methods used in this study are listed below.

#### Dataset preprocessing

The framework preprocesses the datasets by initially eliminating rows with missing values to maintain data integrity. It encodes categorical target labels into a numerical representation with a LabelEncoder. To prevent feature supremacy, feature scaling enables us to place all continuous features on the same scale. The features are normalized with Standard Scaler to achieve a mean of 0 and a standard deviation of 1. This indicates that the input data adheres to the Gaussian distribution, enhancing model performance and accelerating convergence. This comprehensive preparation phase readies the dataset for efficient model training.

1 $${X}_{scaled}=\frac{X-\mu }{\sigma }$$

whereby ${X}_{scaled}$: represents the scaled feature value, $X$: denotes the original feature value, $\mu$: signifies the mean of the feature in the training set, and $\sigma$: standard deviation of the feature in the training set.

#### Avoiding class imbalance issues

To overcome class imbalance difficulties, we used the most popular synthetic minority over-sampling approach (SMOTE) to generate synthetic cases of the minority classes. This is beneficial in achieving dataset balance and enhancing classifier performance, mainly when the minority class is marginalized. In our research, we used Algorithm 2 to handle class imbalance by generating synthetic samples for the minority class. It does this by considering the 2 nearest neighbors for creating these synthetic samples, and automatically balances the dataset. The result is a new, balanced dataset (X_resampled, y_resampled) where the minority class has been artificially increased to match the size of the majority class. This preparation pipeline optimizes the datasets for machine learning models by assuring data quality, standardization, and balanced class distributions.

### Feature selection and feature reduction

To properly predict the desired response, machine learning approaches need feature selection to find the most important features or higher-order attributes^43^. The main goal of feature selection techniques is to reduce the number of attributes to the most significant ones for machine learning algorithm model construction. Importantly, feature selection and feature extraction are different. The use of feature selection before training a machine learning algorithm has the advantage of decreasing the degree of dimensionality of the dataset, resulting in a decrease in the time required to construct a machine learning model. Additionally, it is important to note that feature selection may enhance machine learning measures, such as accuracy and precision^44^. Several feature selection methods exist, including lasso regression and stepwise forward and backward selection^45–48^. Due to its prominence in related research, we chose Mutual Information (MI) for this study.

*Feature selection*

A concise summary of each of the feature selection techniques implemented in our framework is presented. The feature selection technique that demonstrated the highest accuracy score and cross-validation was mutual information (MI). A thorough description of this technique is provided at the end of this subsection.

Correlation-based: This strategy is employed for selecting features in Machine Learning classification jobs. It independently examines every feature to determine its association with the associated class. The features are subsequently sorted according to their correlation values^49^. Information Gain: An indicator of how much information a feature provides regarding a class is called Information Gain (IG)^47^. It calculates the decrease in entropy, which is an indicator of unpredictability and information associated with random variables. The IG metric quantifies the significance of an attribute and its utility in identifying learning classes^43^. In this case, the number that serves as a cutoff for the selected feature in information gain (IG) is called a threshold. The threshold value may be set separately or using 0.05. Employing mathematical calculations, Tsai and Sung determined the final feature threshold value by averaging the frequency^50^. According to Tsai, the standard deviation may be used to estimate the threshold value^51^. Mutual Information: It measures the level of information acquired about one random variable from another. Within the context of feature selection, it assesses the interdependence between a feature X and the target variable Y. The mutual information $MI\left(X;Y\right)$ between a feature X and the target variable Y is defined as:

2 $$MI\left(X;Y\right)=\sum_{x\in X}\sum_{y\in Y}p\left(x,y\right)\mathit{log}\left(\frac{p\left(x,y\right)}{p\left(x\right)p\left(y\right)}\right)$$

where: $p\left(x,y\right)$ is the joint probability of X and Y, $p\left(x\right)$, and $p\left(y\right)$ are the marginal probabilities of X and Y, respectively

Entropy of X:

3 $$H\left(X\right)=-\Sigma p\left(x\right)\mathit{log}p\left(x\right)$$

Entropy of Y:

4 $$H\left(Y\right)=-\Sigma p\left(y\right)\mathit{log}p\left(y\right)$$

Joint Entropy of X and Y:

5 $$H\left(X,Y\right)=-\sum_{x\in X}\sum_{y\in Y}p\left(x,y\right)\text{log}p(x,y)$$

Mutual Information:

6 $$MI\left(X;Y\right)=H\left(X\right)+H\left(Y\right)-H(X,Y)$$

**Fig. 2:**
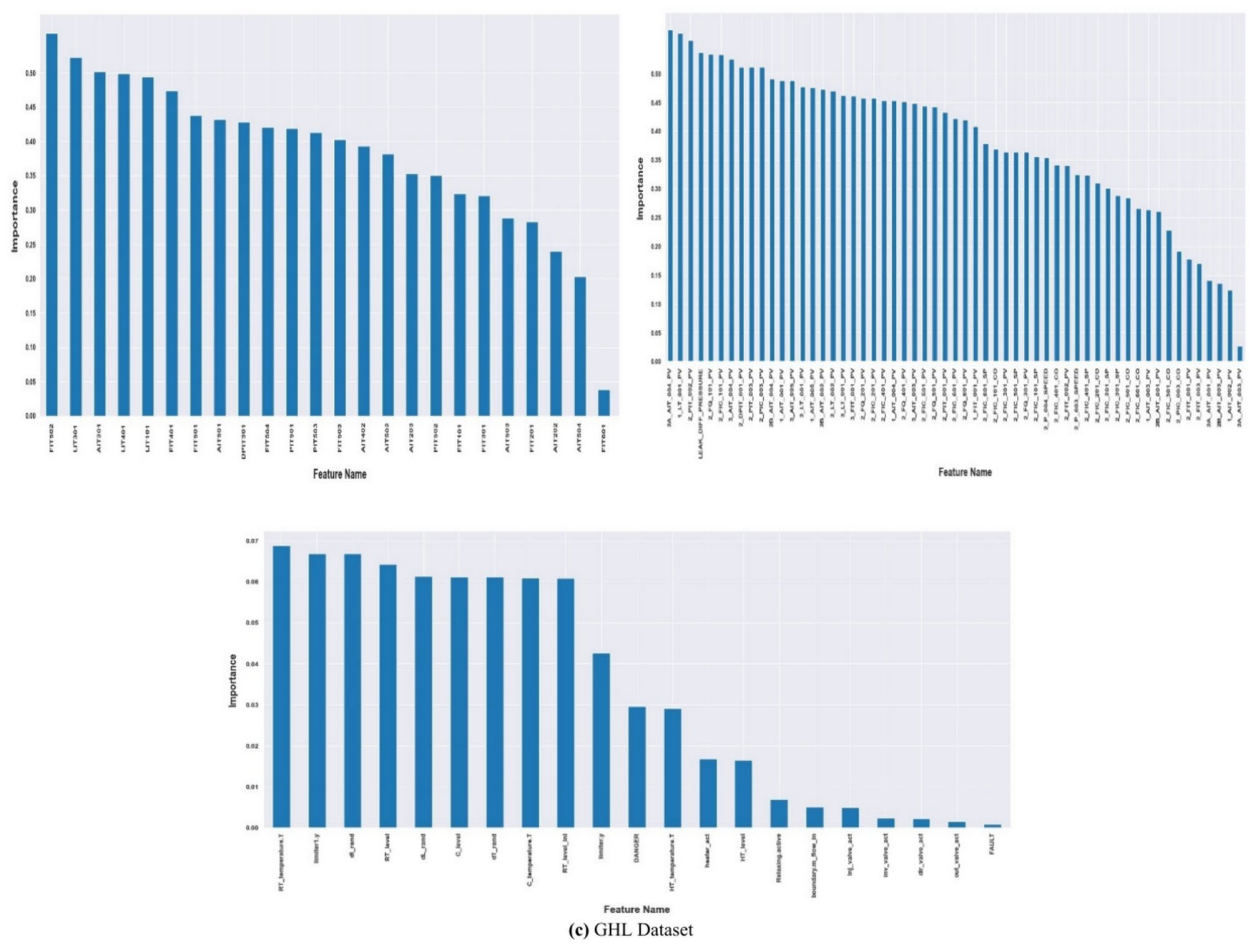
Mutual Information showing the features with the highest and lowest importance scores based on the threshold values of 0.2073, 0.1770 and 0.0109 for the three datasets.

Feature relevance depends on how closely they correlate with the dependent variable in each feature selection method. Figure 2–c show the features in the three datasets with the highest feature selection scores. First ${\text{X}}_{\text{train}}={\mathbb{R}}^{\text{m}*\text{n}}$ be the training dataset with m samples and n features. Then the code conducts the feature selection by computing the Mutual Information between the features (X_train) and the target variable (y_train). Then it uses a Decision Tree Classifier (DTC) with cross-validation (CV) to determine the best threshold (${\text{T}}_{\text{best}}$) in selecting the features with significant importance, aiming to maximise the model’s accuracy. Subsequently, it identifies the most optimal features ${(\text{S}}_{\text{best}})$ when ${\text{S}>\text{S}}_{\text{best}}$, and prepares the training X_train_selected, validation X_val_selected, and test (X_test_selected) datasets for further modelling stages. In our research, we used Algorithm 3 to select feature by computing the Mutual Information between the features (X_train) and the target variable (y_train). It then determines the appropriate threshold using cross-validation. It selects the features ${(\text{S}}_{\text{best}})$ when ${\text{S}>\text{S}}_{\text{best}}$ that yields the highest model performance using the threshold (${\text{T}}_{\text{best}}$). The way we tuned our feature selection technique is shown in Table 2. In addition, the pseudocode for the for the mutual information (MI) feature selection technique is stated in Algorithm 1.

**Algorithm 1:**
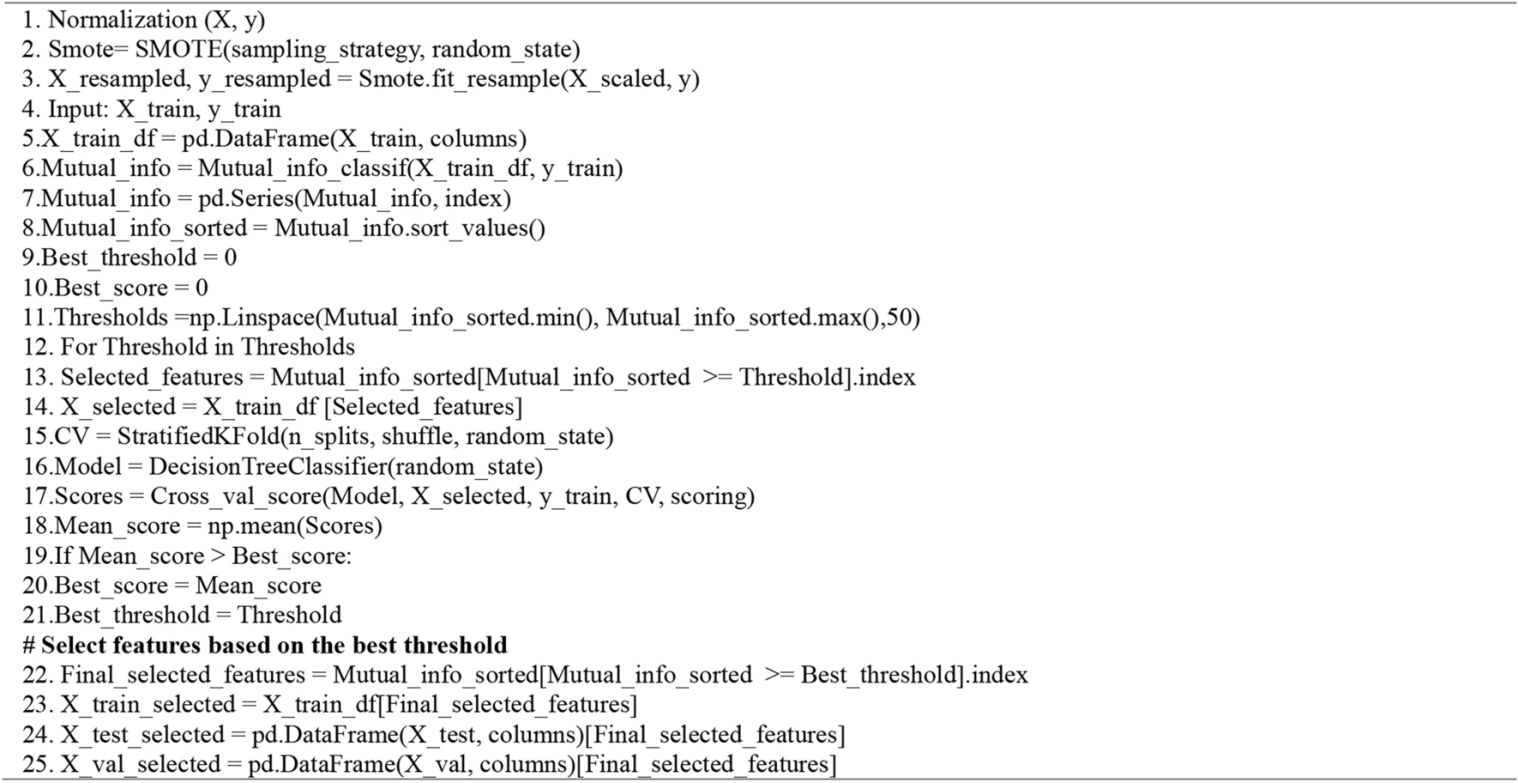
Mutual_Information_Feature_Selection

**Table 2:** Hyperparameter tuning inside the mutual information (MI).

Hyperparameter	Value(s)
Ascending	False
n_splits	5
shuffle	True
random_state	42
Cv	Cv

*Feature reduction*

#### Formulation of PCA and sparse PCA

The essential formulae of PCA and sparse PCA are covered in this section. Ningmin and Jing^52^ extensively analyze formulations for Principal Component Analysis (PCA) and its sparse variations. Principle Component Analysis (PCA) finds linear combinations of the original variables, called principal components, that encapsulate the data’s largest variation. This process may be done using eigenvalue decomposition if A is a covariance matrix or single value decomposition (SVD)on a data matrix A. Summary of the concept is as follows.

Let *n* be independent and identically distributed data items, designated as X_1_, X_2_,…, X_n_, be observed on *p* variables. The potentially vast amount of *p* makes Principal Component Analysis (PCA) a good way to reduce its dimensionality. Consider a population covariance matrix $\Sigma ={\mathbb{E}}\left(X{X}^{T}\right)$ where ${\mathbb{E}}\left(X\right)=0$. The eigenvalue decomposition of a matrix *∑* may be expressed in the following manner:

7 $$\Sigma ={\lambda }_{1}{v}_{1}{v}_{1}^{T}+{\lambda }_{2}{v}_{2}{v}_{2}^{T}+\dots +{\lambda }_{p}{v}_{p}{v}_{p}^{T}$$

$${\lambda }_{1}\ge {\lambda }_{2}\ge {\lambda }_{p}\ge 0, \left(eigen values\right)$$

$${V}_{i}^{T}{V}_{j}={\delta }_{ij} , \left(eigen vectors\right)$$

using the “ideal” d-dimensional mapping $X\to {\Pi }_{d}X$,

Specifically, we may define the sample covariance matrix as:

8 $$\widehat{\Sigma }={n}^{-1}\left(X,{X}_{1}^{T}+\dots +{X}_{n}{X}_{n}^{T}\right)$$

We calculate $\left({\widehat{\lambda }}_{j},{\widehat{v}}_{j}\right)$ using $\widehat{\Sigma }$ eigenvalue decomposition where.

9 $${\widehat{V}}_{d}=\left({\widehat{v}}_{1},\cdots ,{\widehat{v}}_{d}\right), {\widehat{\Pi }}_{d}={\widehat{V}}_{d}{\widehat{V}}_{d.}^{T}$$

The significance of PCA lies in its ability to minimize component count. Initial identification of the dataset’s most important variances allows primary elements to minimize data while retaining critical information. Second, factual analysis is easier since the significant segments (primary components) are uncorrelated. Naturally, Principal Component Analysis (PCA) has downsides. We are driven by the requirement that the major components be linear blends of all the original variables (see, e.g.^53^).

#### Formulation of PCA

Consider the input data matrix $X={\left[{x}_{1},{x}_{2,\dots }{x}_{n}\right]}^{T}\in {R}^{n\times d}$ , where *n* is the size of the observed data, and *d* represents its dimensionality. Assume all the variables are centered, i.e., ${\Sigma }_{i}{x}_{i}=0$, and $\Sigma =\frac{1}{n}{X}^{T}X\in {R}^{d\times d}$ be the data covariance matrix. PCA identifies *p* <  < *d* linear combinations with *n* parameters in a projected linear space. These linear combinations are represented as: ${\widetilde{z}}_{k}={X}^{T}{u}_{k}=\sum_{i=1}^{d}{u}_{k,i}{x}_{i}$, where ${\widetilde{z}}_{k}$ denotes the *k*th principal component (PC) and *u*_*k*_ represents the unit-length loadings vector. PCA may be done using eigenvalue decomposition of the covariance matrix or singular value decomposition. PCA may be derived by maximizing data variance. Finding *u* that optimizes input data variance is the goal. This creates an optimization difficulty^52^:

10 $$\underset{u}{max}{ u}^{T}\Sigma u s.t. \Vert u\Vert =1$$

#### Formulation of sparse PCA

Sparse Principal Component Analysis (SPCA), sometimes known as sparse PCA, is an advanced statistical analysis method for multivariate data sets. SPCA aims to produce sparse eigenvectors by forcing a portion of lower-importance loadings to be zero. Several ways use a constraint or penalty term in Principal Component Analysis (PCA) to achieve high sparsity in retrieved components (1). The ${l}_{0}$-norm is used to construct sparse PCA as a constrained minimization problem^52^.

11 $$u=arg\underset{u}{max}{ u}^{T}\Sigma u s.t. {\Vert u\Vert }_{2}=1,{\Vert u\Vert }_{0}\le k$$

where *k* is the number of non-zero loadings. The SPCA problem in (2) is non-convex and NP-hard. The numerous formulations and methodologies fall into three categories: data-variance-maximization, minimal-reconstruction-error, and probabilistic modelling.

Two interrelated elements must be considered while converting from PCA to SPCA: (1) correlations between values and/or loads in distinct components and (2) constituent variability within the row space represented by the initial data.

Sparse PCA eliminates the first factor—calculating scores, residuals, and variance in PCA. Captured variance is a key indicator of model quality and change analysis. So, precise calculation is essential. The second factor affects interpretability, especially in multi-part models.

To improve Principal Component Analysis (PCA), sparse-inducing limitations or penalties like ${l}_{0}$ or ${l}_{1}$ norms may be used in most SPCA calculations. To simplify the LASSO approach^53^, created the SCoTLASS algorithm, which incorporates the least total shrinkage and selection operator^53^. It penalizes loadings employing the $l_1$ norm (absolute value). The SCoTLASS requirements are:

12 $${\widehat{P}}^{SL}=\mathit{arg}\underset{p}{max}{\Vert {X}_{p}\Vert }_{F}^{2} s.t. {\Vert p\Vert }^{1}\le {c}_{1}{\Vert p\Vert }_{2}^{2}=1$$

Sparse loading is represented as $\hat{P}^{{SL}},$ with the resulting sparse loading denoted by *SL*. The SCoTLASS optimization approach requires orthogonality between the second and subsequent sparse loadings and the remaining components to achieve the remaining components. SCoTLASS criterion (3) could require large computational resources, making their application in information analysis unusual^54^. Similarly, the lasso constraint limits the amount of non-zero components in $\hat{P}^{{SL}},$ due to the number of observations in the data. In our research, we used the approach outlined in Algorithm 2. Our research reverts the prediction to the original feature space, distinguishing it from previously described techniques.

It initially reduces the dataset into a specific count of principal components, thereby reducing the original feature space to a more informative, smaller set of features. This initializes a SparsePCA model to condense the feature space to the precise number of principal components. Determines the precise number of principal components by employing the selected training parameters. The principal components are projected into the new lower-dimensional space by selected training parameters. The transformation method guarantees consistency across the training, validation, and test sets by applying the transformation that was previously learnt to selected validation, and test parameters. In addition, the pseudocode and the hyperparameter tuning for the SparsePCA feature reduction technique is stated in Algorithm 2.

**Algorithm 2:**
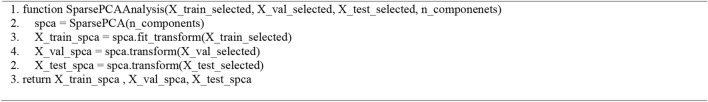
Function SparsePCA

### Cyberattack detection method

This study suggests attention-driven deep neural network models in conjunction with the multitude of feature selection, and feature reduction strategies to increase the classification accuracy of cyber-attacks (such as noise and spoofing attacks) detection on ICS. The proposed approach aims to identify and mitigate cyberattacks occurring at the physical layer via continuous monitoring and analysis of system behaviour. The overall process of cyberattack detection, based on the suggested interdisciplinary framework, is outlined via a pipeline as follows.

#### Attention mechanism

The attention process directs its focus towards the most pertinent segments of an input sequence by giving distinct priority values (attention weights) to each time step. This enables the model to efficiently capture extensive relationships and intricate patterns in sequential data, resulting in enhanced performance in language modelling, translation, and time series analysis. Given input $X\in {\mathbb{R}}^{T*F}$ (where T represents a count of time steps and F is the number of features), the attention layer computes:

13 $$logits=X.W$$

where $X\in {\mathbb{R}}^{F*1}$ is a trainable weight matrix.

Calculating attention weights and performing a weighted sum of the inputs and outputs of the attention layer using the equations.

14 $$attention\_weights=softmax\left(logits, axis=1\right)$$

15 $$weighted\_sum=\sum_{t=1}^{T}\left(attention\_weights\cdot {X}_{t}\right)$$

16 $$output=weighted\_sum$$

The final output of the attention layer is the weighted sum of the input sequence, governed by these attention weights.

#### Deep learning models

After receiving data from the *X* sensor of dimension *d*, at time *t*, with attention weights, the outcomes of the suggested framework models are categorized as normal or malicious. After the models have undergone sufficient training, they may be used to categorize new data samples. The training, test and validation datasets have been created for model(s) verification. Table 3 shows how we tuned our deep learning models employed in the framework.

**Table 3:** Hyperparameter tuning inside the framework.

Parameter	Value(s)
Language	Python
Libraries	Pandas, Numpy, Matplotlib, Sckitlearn, Matplotlib, Keras, and Scipy
Train set	60%
Test set	20%
Validation set	20%
RNN, LSTM, Bi-LSTM layers	[2]
Neurons per RNN, LSTM, Bi-LSTM layers	[128,64,32,1]
Attention Layer	[1]
Dropout	[0.2]
Dense layers	[2]
Neurons per dense layer	[128,1]
Activation function	[Relu, Sigmoid]
Optimizer	Adam
Initial learning rate	0.1
Final learning rate	0.001
Number of Epochs	100
Batch size	64

It includes Deep RNN, Deep LSTM, and Deep Bi-LSTM models. We used these models to improve accuracy and reduce computational complexity when detecting cyberattacks on ICSs. Of these three models, Deep LSTM model outperformed the Deep RNN and Deep Bi-LSTM models in outcomes and exhibited lesser training times.

##### Recurrent neural networks (RNN)

Recurrent Neural Networks (RNN) exhibit high suitability for analysing and predicting time series data. Their ability to capture patterns and temporal relationships makes them valuable for detecting anomalies and forecasting. The explored deep neural networks use historical data and feature selection to overcome the nonlinearity issue to find appropriate features.

The RNN model consists of four layers. The first layer, the dense layer, consists of 128 units; the second one, the simple RNN layer, consists of 64 units; the third one, the second simple RNN layer, consists of 32 units; and the last layer, the second dense layer, consists of 1 unit only. Between the four layers, we applied the dropout regularization rate of 0.2 to allow the model to overcome the overfitting problem.

The RNN model consists of four layers. The first layer, the dense layer, consists of 128 units; the second one, the simple RNN layer, consists of 64 units; the third one, the second simple RNN layer, consists of 32 units; and the last layer, the second dense layer, consists of 1 unit only. Between the four layers, we applied the dropout regularization rate of 0.2 to allow the model to overcome the overfitting problem.

The following determines the RNN model’s outputs ${y}_{t}^{RNN}$ and hidden layers ${h}_{t}^{RNN}$ for a given feature vector {${x}_{1}^{RNN}$, ${x}_{2}^{RNN}$, …${x}_{t}^{RNN}$}:

17 $${h}_{t}^{RNN}={g}_{h}\left({w}_{i}{x}_{t}^{RNN}+{w}_{R}{h}_{t-1}^{RNN}+{b}_{h}\right)$$

18 $${y}_{t}^{RNN}={g}_{y}\left({w}_{y}{h}_{t}^{RNN}+{b}_{y}\right)$$

The weighted matrices ${w}_{i}$, ${w}_{R}$, ${w}_{y}$ represent the connections between a neural network’s input, output, and hidden layers. The bias vectors ${b}_{h}$ and ${b}_{y}$ are additional parameters used to adjust the network’s outcome. The variable t denotes the count of samples, while r represents the chosen activation function. The activation functions that may be considered are Rectified Linear Unit (ReLU) and sigmoid.

##### Long short-term memory (LSTM)

LSTM mitigates the issue of vanishing gradients present in conventional RNNs and is explicitly engineered to retain long-term dependencies in time-series data. The LSTM model includes four layers. It solves the problem of gradient vanishing. The first layer the dense layer consists of 128 units, the second one the LSTM layer consists of 64 units, the third one the second LSTM layer consists of 32 units, the last layer the second dense layer consists of 1 unit only, and the dropout follows the same structure as discussed above.

The model of the LSTM has three multiplication components, namely the input gate ${i}_{t}^{LSTM}$, forget gate ${f}_{t}^{LSTM}$, and output gate ${o}_{t}^{LSTM}$. These gates play a crucial role in regulating the cell ${c}_{t}^{LSTM}$, which is responsible for storing both past and future knowledge.

The components are determined by the following calculation:

19 $${f}_{t}^{LSTM}=\sigma \left({w}_{f}\left[{h}_{t-1}^{LSTM},{x}_{t}^{LSTM}\right]+{b}_{f}\right)$$

20 $${i}_{t}^{LSTM}=\sigma \left({w}_{i}\left[{h}_{t-1}^{LSTM},{x}_{t}^{LSTM}\right]+{b}_{i}\right)$$

21 $${\widetilde{c}}_{t}^{LSTM}=\mathit{tan}h\left({w}_{c}\left[{h}_{t-1}^{LSTM},{x}_{t}^{LSTM}\right]+{b}_{c}\right)$$

22 $${c}_{t}^{LSTM}={f}_{t}^{LSTM}*{c}_{t-1}^{LSTM}+{i}_{t}^{LSTM}*{\widetilde{c}}_{t}^{LSTM}$$

23 $${o}_{t}^{LSTM}=\sigma \left({w}_{0}\left[{h}_{t-1}^{LSTM},{x}_{t}^{LSTM}\right]+{b}_{0}\right)$$

24 $${y}_{t}^{LSTM}={o}_{{t}^{*}}^{LSTM}\mathit{tan}h\left({c}_{t}^{LSTM}\right)$$

where the variable “*I*” represents an input gate, “*f*” represents a forget gate, “*o*” represents an output gate, and “*c*” represents a cell activation. The weight matrices are denoted as ${w}_{i}$, ${w}_{f}$, ${w}_{o}$, and ${w}_{c}$. The feature vector is denoted as {${x}_{1}^{LSTM}$, ${x}_{2}^{LSTM}$, …${x}_{t}^{LSTM}$}, the output of LSTM is represented as ${y}_{t}^{LSTM}$, the previous hidden states are denoted as ${h}_{t-1}^{LSTM}$, and the bias vectors are represented as ${b}_{i}$, ${b}_{f}$, ${b}_{c}$, and ${b}_{o}$.

##### Bi-directional long short-term memory (Bi-LSTM)

Bi-LSTM augments this functionality by analyzing input data in both forward and backward orientations, hence allowing the model to identify more intricate patterns in sequential data. Bi-directional Long Short-Term Memory (Bi-LSTM) also follows the same layer and dropout structure as the first layer: the dense layer consists of 128 units, the second one being the Bi-LSTM layer consists of 64 units, the third one $\widehat{y}$ the second BiLSTM layer consists of 32 units, while the last layer, the second dense layer, consists of 1 unit only.

Bi-directional Long Short-Term Memory (Bi-LSTM) models include the integration of two distinct Recurrent Neural Networks (RNNs). This architectural design enables the neural networks to possess bidirectional information on the sequence at each temporal iteration. The Bi-LSTM model employs the same mathematical procedures as the RNN model, with the distinction that the output layer ${\widehat{y}}_{t}^{BiLSTM}$ at time t encompasses the activations from both the forward and backward directions^55^.

25 $${\overrightarrow{h}}_{t}^{RNN}=f\left(\overrightarrow{w}{x}_{t}^{RNN}+\overrightarrow{v}{\overrightarrow{h}}_{t-1}^{RNN}+\overrightarrow{b}\right)$$

26 $${\overleftarrow{h}}_{t}^{RNN}=f\left(\overleftarrow{w}{x}_{t}^{RNN}+\overleftarrow{v}{\overleftarrow{h}}_{t+1}^{RNN}+\overleftarrow{b}\right)$$

27 $${\widehat{y}}_{t}^{BiLSTM}=g\left(u\left[{\overrightarrow{h}}_{t}^{RNN},{\overleftarrow{h}}_{t}^{RNN}\right]+c\right)$$

The outputs of Deep RNN, Deep LSTM, and Deep Bi-LSTM models are examined to forecast the accuracy of detecting cyber-attacks on Industrial Control Systems (ICS). This is accomplished via a softmax layer, and the performance of the models is assessed using numerous evaluation metrics.

**Fig. 3:**
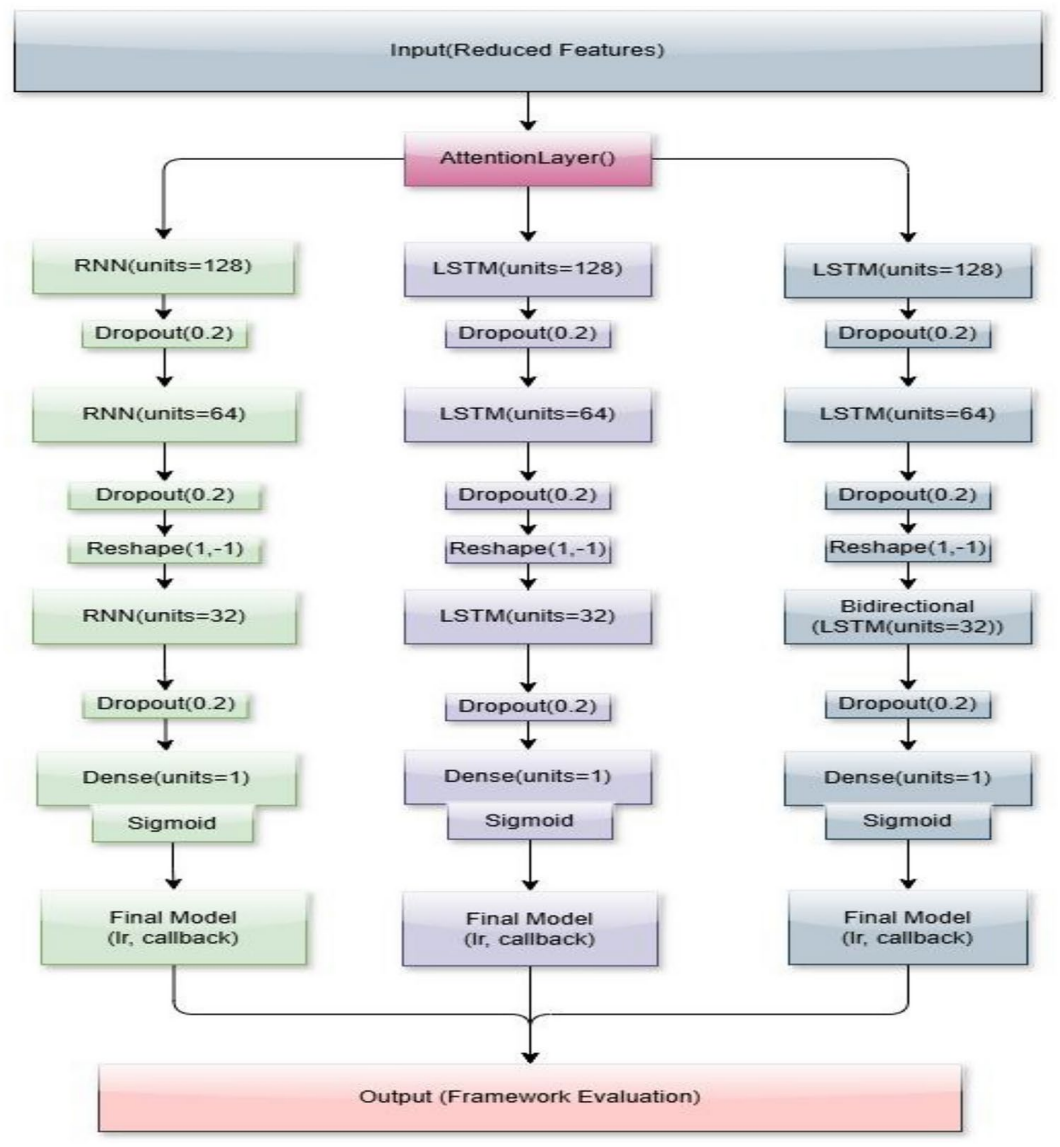
Proposed framework’s schematic representation.

Consequently, the outputs of the Deep RNN, Deep LSTM, and Deep Bi-LSTM models are converted into dimensions suitable for the dense layers. The initial dense layer comprises 128 units and employs the ReLU activation function. The subsequent dense layer has 64 units, succeeded by a ReLU activation function. The third dense layer has 32 units and is succeeded by a ReLU activation function. The fourth dense layer comprises a single unit, succeeded by a sigmoid activation function. Ultimately, the conclusive model with a learning rate and verbosity forecasts the result. The structure of the proposed framework for detecting cyberattacks in ICSs is shown in Fig. 3. Figure 3 illustrates the comprehensive design of the suggested framework. The pseudocode for the Deep RNN, Deep LSTM, and Deep Bi-LSTM models is shown in Algorithm 3. This renders them very proficient in identifying cyber-attacks that display complex temporal correlations, prevalent in ICS settings.

The overall computational complexity of your model is ascertained by the aggregate of the complexities of each layer and the attention layer. The following is a detailed explanation of the connectivity between all the elements:

28 $$T(n)=O\left(n\times \mathit{max}\left({u}_{1}^{2},{u}_{2}^{2},d\right.\right)$$

This reduces to the principal component that increases most rapidly with n, the input size.

If the model is dominant, then $T(n)=O\left(n\times {u}_{1}^{2}\right)$; otherwise, if Attention is dominant, then $T(n)=O\left(n\times d\right)$.

The computational cost of our model is encapsulated in this complexity statement in terms of input size n, model units u, and attention layer dimensions d.

In our research we utilized Algorithm 3 which provides a step-by-step procedure for data preparation, constructing a sequential model (potentially incorporating dense, recurrent, and attention processes), training the model with an adaptable learning rate, and assessing its performance on test data using metrics such as the confusion matrix and z-score. The strategy is specifically developed to effectively process sequential data and incorporates techniques such as dropout and learning rate scheduling to mitigate the risk of overfitting.

**Algorithm 3:**
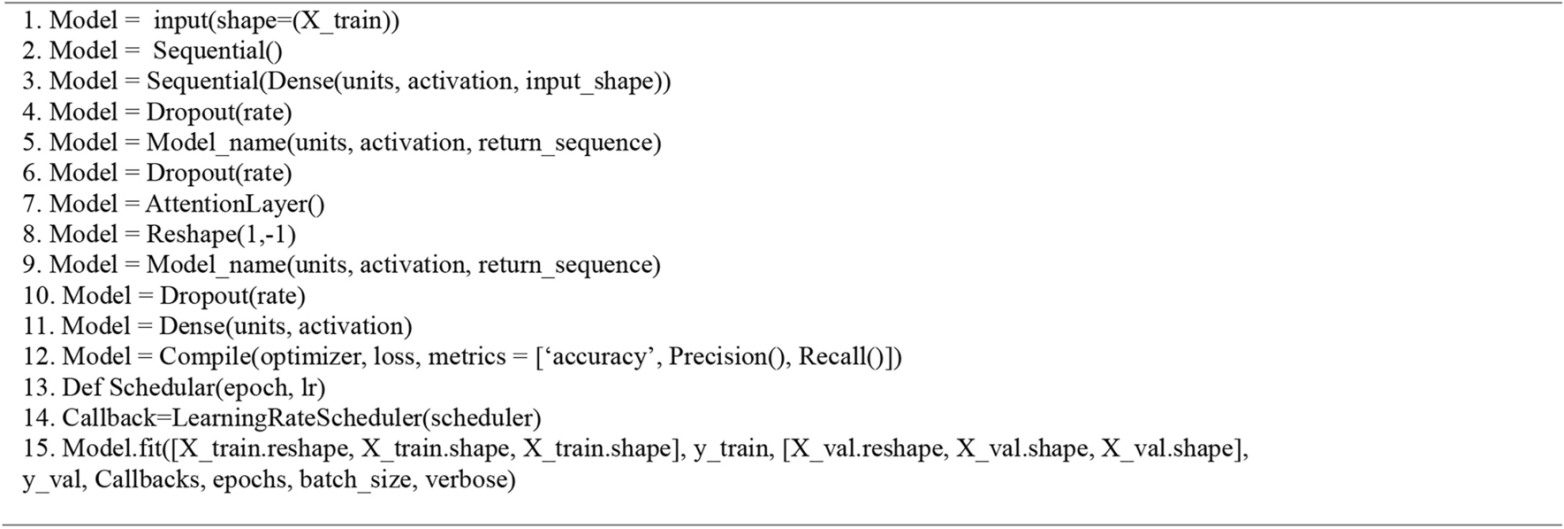
Deep RNN/LSTM/BiLSTM Models

### Anomaly detection method

This stage details the steps to choose, finetune, and train the best anomaly detection model. This concerns the adjustment of the threshold for detecting anomalies. In this context, we propose to use a statistical methodology employed in several scholarly publications^19,29^. To begin with, the variance between the expected result, denoted as $,$ generated by the neural network, and the actual outcome, referred to as *y*, is calculated as:

29 $$\text{e}=\left|\text{y}-\widehat{\text{y}}\right|$$

The variance is calculated for the training dataset. Subsequently, the given data’s mean, µ_e_, and standard deviation, σ_e_, are computed. Subsequently, the variance in the validation dataset, *e*_*v*_, is computed. At this juncture, we preferred to use the validation dataset split during the pre-processing dataset stage. Meanwhile, the mean and standard deviation derived from the training dataset are then used to calculate a z-score for the validation dataset, denoted as *z*_*e*_, similarly for the testing dataset as well. The formal definition is given in Eq., 28.

30 $${z}_{e}=\frac{{e}_{v}-{u}_{e}}{{\sigma }_{e}}$$

**Fig. 4:**
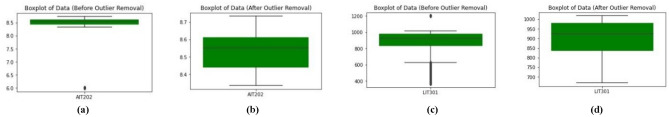
Box plots to visualize the outliers before and after their removal in the SWaT dataset from the AIT 202 and LIT301 sensors.

**Fig. 5:**
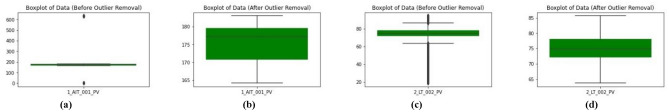
Box plots to visualize the outliers before and after removal in the WADI dataset from the 1_AIT_001_PV and 2_LT_002_PV sensors.

**Fig. 6:**
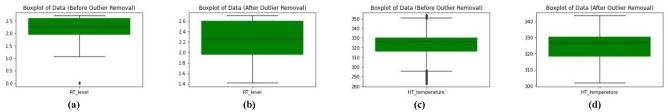
Box plots to visualize the outliers before and after removal in the GHL dataset from the RT_level and HT_temperature sensors.

We preferred to calculate the individual thresholds for each attribute based on them to determine the threshold. respective maximum z-scores as shown in Figs. 4, 5 and 6. To identify anomalies within the test dataset, the z-score is computed for said dataset, using the mean and standard deviation derived from the training dataset. In the case of a particular sample, the identification of an anomaly occurs when the z-score of any given characteristic is above a predetermined threshold^30^.

## Experimental results

This section presents a comprehensive overview of the experimental datasets, the evaluation criteria, the resulting outcomes, and the comparison with the existing approaches based on the precision, recall/detection rate, and f1-score, to assess the efficacy of the proposed framework.

### Dataset description

To conduct a more comprehensive assessment of the suggested framework, it is necessary to get genuine datasets that include benign ICS activity and malicious ICS behaviour. For experiments, the dataset must possess enough malicious samples. In this context, we took into account the three industrial entities: SWaT dataset^38,39^, the WADI dataset^40,56^, and the GHL dataset^41,42^.

We evaluated our framework on three publicly available datasets frequently utilized for ICS research.

**SWaT-**The Singapore University of Technology and Design is the site of the collection of the SWaT (secure water treatment) dataset^38,39^. Researchers see this raw water treatment facility, often used in public infrastructure, as a testbed for developing different cyberattack detection techniques. Its construction included four days of attack activity and seven days of regular system operation using sensors, actuators, PLC I/O signals, and human–machine interfaces (HMIs). There are 26 actuators and 25 sensors in the system. Each of these features is essential to the operation of the plant and acts as a warning sign before anything goes wrong. They aid in preventing costly equipment damage. Along with details on the regular functioning of the system, SWaT includes information on 36 attack scenarios, such as an attack on an undersized tank through pump shutdown, damage to the pump, and more^25^. The SWaT dataset consists of a total of 36 assaults, which may be categorized as follows: 26 attacks are classified as single-stage single-point (SSSP) attacks, 4 attacks fall under the category of single-stage multi-point (SSMP) attacks, 2 attacks are classified as multi-stage single-point (MSSP) attacks, and finally, 4 attacks are categorized as multi-stage multi-point (MSMP) attacks^25^. The SWaT dataset contains a total of 449,919 samples.

**WADI-**Collected from a reduced-scale water distribution testbed and assembled by SWaT developers^56^. The dataset records the functioning of the testbed over 16 days, with 59 sensor observations and 45 actuator signals. Additionally, the dataset comprises the control signals of seven actuators and their respective setpoints. The dataset comprises a total of 15 deliberate attacks that were introduced throughout the last two days of the testbed operation^57^. These attacks specifically targeted the various components of the cyber-physical system, with the primary objective of disrupting the water delivery to the consumer tanks. The experiments were carried out via the manipulation of valve openings and the spoofing of sensor readings^6^.

**The GHL-**Gasoil heating loop (GHL) dataset^41^ was compiled at the Future Technologies Kaspersky Lab. The experimental setup comprises three reservoirs, namely a receiving tank (RT), a heating tank (HT), and a collection tank (CT). A fraction of the gasoil mixture originating from the RT unit is conveyed to the HT unit to undergo heating. Subsequently, the heated mixture is reintroduced back into the RT unit, and this cyclic process is iterated. The procedure is conducted iteratively until the temperature of the combination at room temperature (RT) achieves the intended value. Subsequently, the heated mixture is transferred to tank CT, while tank RT is filled with an additional portion of the gasoil combination. The gasoil setup model has samples corresponding to different features associated with the process. There are a total of 19 attributes in the GHL dataset. The occurrence of malicious system behaviour was noticed because of cyber-attacks. The maximum RT level, maximum HT temperature, pump frequency, and system relaxing time value are among the ones that may be changed without authorization^41^. The attack on the RT overflow is distinguished by an elevated real RT level. In contrast, the transferring issue resulting from an RT valve failure is distinguished by a negligible actual RT level.

### Evaluation metrics

The efficacy of our suggested framework is evaluated using the widely known performance measures of accuracy, precision, recall, F1-score, and so on in the research associated with cyber-attack detection in ICS. These metrics were selected due to their ability to provide a thorough evaluation of the model’s performance, especially in unbalanced classification scenarios, prevalent in network intrusion detection. They guarantee a comprehensive assessment by factoring in both false positives and false negatives, which are essential for real-time detection in Industrial Control Systems (ICS).

1. Accuracy: The term refers to the ratio of accurate predictions made by the model concerning the overall number of predictions:
  31 $$\text{Accuracy }= \frac{\text{TP }+\text{ TN}}{\text{TP}+\text{TN }+\text{FN }+\text{ FN}}$$
2. Precision (PPV): The “*Positive Predictive Value (PPV)*”*,* quantifies the accuracy of the projected positive cases:
  32 $$\text{PPV}=\frac{\text{TP}}{\text{TP}+\text{FP}}$$
3. Recall (DR) / Sensitivity (TPR): The classifier’s *‘True Positive Rate’ (TPR),* it quantifies the number of true positive cases accurately detected by the model:
  33 $$\text{TPR}=\frac{\text{TP}}{\text{TP}+\text{FN}}$$
4. F1 – Score: Represents a geometric mean between *‘precision’,* and *‘recall’,* with a sign of 1 indicating perfect accuracy and recall:
  34 $$\text{F}1-\text{Score}=\frac{2 \times \text{ PPV }\times \text{ TPR}}{\text{PPV}+\text{TPR}}$$
5. Log Loss: Additionally, log loss is employed to measure how well learning algorithms are working:
  35 $$\text{Log Loss}=\frac{{-\Sigma }_{\text{y}=1}^{\text{j}}{\Sigma }_{\text{x}=1}^{\text{n}}\text{f}(\text{x},\text{y})\text{ log}(\text{p}(\text{x},\text{y}))}{\text{n}}$$
  where the range of classes is j, the number of observations is n, and the probability that observation *x* belongs to class *y* is *f*(*x, y*)^58^. The process of minimizing log loss, which is often referred to as “cross-entropy, involves optimizing the logarithmic probability of seeing data from a model^58^”.
6. Receiver Operating Characteristics (ROC)- Area Under the Curve (AUC): ROC is a graphical representation that demonstrates the diagnostic capability of a binary classifier when the discrimination threshold is altered.
  36 $$ROC AUC=\underset{0}{\overset{1}{\int }}dtTPR\left({FPR}^{-1}\left(t\right)\right)$$

The AUC quantifies the level of distinguishability exhibited by the model. It measures the model’s ability to differentiate between different classes. A receiver operating characteristic (ROC) curve with an area under the curve (AUC) of 1 signifies flawless discrimination. In contrast, an AUC of 0.5 suggests no discrimination (i.e., the model’s accuracy is equivalent to random chance).

### Experimental description

The experiments were conducted using a Microsoft Windows 11 Pro device with an Intel(R) Core (TM) i7-10610U CPU @ 1.80 GHz, operating at a frequency of 2304 MHz. The device had 4 cores and 8 logical processors, 32 GB RAM and a 512 GB SSD. The framework has been evaluated using the Python 3.11.4 (64-bit) version, employing several libraries such as Numpy, Pandas, Matplotlib, Keras, Scipy, and SkLearn.

The efficacy of the suggested framework for identifying cyberattacks in industrial control systems (ICSs) that integrate the benefits of attention-driven Deep RNN, Deep LSTM, and Deep Bi-LSTM neural networks with the MI and SPCA was assessed via experiments carried out on the three datasets. The datasets are partitioned into a training set comprising (60%) of the data, a test set comprising (20%) of the data, and the remaining (20%) of the validation data. Adam and ReLU were thought to be optimizers and activation functions respectively. Initially, the framework underwent evaluation on the complete dataset for cyber-attack detection on the Industrial Control Systems (ICS). Subsequently, the framework was assessed for its ability to detect different attack categories in diverse scenarios. This evaluation involved utilising various loss functions to examine their susceptibility to outliers. The loss functions employed for this purpose were: mean squared error (MSE), mean absolute error (MAE), LogCosh, Huber, and root mean squared error (RMSE). The experimental procedure included conducting 100 epochs with a dropout rate of 0.2 and a batch size of 64.

The findings for the SWaT, WADI, and GHL datasets are shown in Tables 4 and 5 respectively.

**Table 4:** Parameter estimation.

Dataset	Learning rate	Training accuracy (%)	Testing accuracy (%)	Training Precision (%)	Testing precision (%)	Training recall (%)	Testing recall (%)	Training F1-score (%)	Testing F1-Score (%)	Z-score
SWaT	0.1	99.73	99.80	99.80	99.80	99.80	99.80	99.80	99.80	− 0.8754
	0.01	99.71	99.78	99.78	99.78	99.78	99.78	99.78	99.78	− 0.9231
	**0.001**	**99.76**	**99.84**	**99.84**	**99.84**	**99.84**	**99.84**	**99.84**	**99.84**	− **0.9355**
WADI	**0.1**	**99.84**	**99.82**	**99.83**	**99.82**	**99.83**	**99.82**	**99.81**	**99.79**	− **0.4351**
	0.01	99.84	99.82	99.83	99.82	99.83	99.82	99.81	99.79	− 0.4320
	0.001	99.72	99.72	99.71	99.70	99.71	99.72	99.70	99.68	− 0.4264
GHL	0.1	**99.80**	**99.82**	99.74	99.82	99.72	99.82	**99.76**	**99.82**	**−∞**
	0.01	**99.80**	**99.82**	99.76	99.83	99.75	99.83	99.74	99.83	**−∞**
	0.001	**99.80**	**99.82**	**99.78**	**99.83**	**99.78**	**99.83**	99.76	99.83	**−∞**

Bold values indicate a particular model performed better compared to others under consideration based on various evaluation metrics.

**Table 5:** Evaluating framework on multiple datasets using multiple feature selection techniques.

Feature selection	Dataset	Algorithm	Precision	Recall	F1-score	Log loss	AUC score	TPR	FPR	Training time in building the model	Testing the model (s)
Mutual information	SWaT	RNN	99.63	99.63	99.63	0.1324	99.63	0.9963	0.0035	18880 s	22 s
		**LSTM**	**99.84**	**99.84**	**99.84**	**0.0563**	**99.84**	**0.9984**	**0.0029**	**12800 s**	**17 s**
		BiLSTM	99.63	99.63	99.63	0.1326	99.63	0.9963	0.0028	14000 s	25 s
		Hybrid RLBiL	99.71	99.71	99.71	0.1028	99.71	0.9971	0.0022	15800 s	44 s
	WADI	RNN	99.71	99.71	99.71	0.1040	99.71	0.9971	0.0004	14800 s	12 s
		**LSTM**	**99.83**	**99.83**	**99.83**	**0.0614**	**99.83**	**0.9983**	**0.0004**	**14022 s**	**10 s**
		BiLSTM	99.80	99.80	99.80	0.0708	99.80	0.9980	0.0007	14866 s	11 s
		Hybrid RLBiL	99.81	99.81	99.81	0.0681	99.81	0.9981	0.0012	19899 s	23 s
	GHL	RNN	99.82	99.82	99.82	0.0613	99.80	0.9982	0.0033	12898 s	7 s
		**LSTM**	**99.83**	**99.83**	**99.83**	**0.0615**	**99.82**	**0.9983**	**0.0031**	**13663 s**	**12 s**
		BiLSTM	99.82	99.82	99.82	0.0645	99.80	0.9982	0.0036	13697 s	12 s
		Hybrid RLBiL	99.94	99.94	99.94	0.0221	99.93	0.9994	0.0033	17780 s	44 s
Correlation coefficient	SWaT	RNN	99.65	99.65	99.65	0.1260	99.65	0.9965	0.0052	11246 s	20 s
		**LSTM**	**99.72**	**99.72**	**99.72**	**0.0991**	**99.72**	**0.9972**	**0.0030**	**11245 s**	**20 s**
		BiLSTM	99.77	99.77	99.77	0.0825	99.77	0.9977	0.0029	11276 s	29 s
		Hybrid RLBiL	99.70	99.70	99.70	0.1073	99.70	0.9970	0.0024	16445 s	41 s
	WADI	RNN	98.27	98.27	98.27	0.6220	98.31	0.9827	0.0310	14402 s	11 s
		**LSTM**	**99.57**	**99.57**	**99.57**	**0.1566**	**99.56**	**0.9957**	**0.0033**	**17206 s**	**18 s**
		BiLSTM	99.06	99.06	99.06	0.3392	99.05	0.9906	0.0136	18407 s	21 s
		Hybrid RLBiL	99.74	99.74	99.74	0.0946	99.73	0.9974	0.0006	14408 s	11 s
	GHL	RNN	98.18	98.18	98.18	0.6562	98.18	0.9818	0.0000	210 s	7 s
		**LSTM**	**98.18**	**98.18**	**98.18**	**0.6562**	**98.18**	**0.9818**	**0.0000**	**200 s**	**5 s**
		BiLSTM	98.18	98.18	98.18	0.6562	98.18	0.9818	0.0000	244 s	6 s
		Hybrid RLBiL	80.43	80.43	80.43	7.0551	80.43	0.8043	0.0000	166 s	44 s
Information gain	SWaT	RNN	87.22	87.22	86.26	4.6055	87.21	0.8722	0.0683	14000 s	10 s
		**LSTM**	**87.54**	**87.54**	**86.56**	**4.4913**	**87.53**	**0.8656**	**0.0618**	**15228 s**	**13 s**
		BiLSTM	87.78	87.78	86.70	4.4119	87.66	0.8670	0.0591	16808 s	17 s
		Hybrid RLBiL	87.47	87.47	86.56	4.5175	87.46	0.8656	0.0680	18619 s	19 s
	WADI	RNN	93.83	93.83	93.33	2.2231	93.84	0.9333	0.0332	17204 s	18 s
		**LSTM**	**89.55**	**89.55**	**89.52**	**3.7682**	**89.57**	**0.8952**	**0.0445**	**16000 s**	**4 s**
		BiLSTM	93.93	93.93	93.78	2.1865	93.94	0.9378	0.0326	16406 s	16 s
		Hybrid RLBiL	89.95	89.95	88.90	3.7682	89.57	0.8890	0.0476	14004 s	10 s
	GHL	RNN	98.78	98.78	98.79	0.04414	98.77	0.9879	0.0238	244 s	6 s
		**LSTM**	**99.32**	**99.32**	**77.06**	**0.2448**	**81.34**	**0.7706**	**0.0221**	**166 s**	**4 s**
		**BiLSTM**	**99.32**	**99.32**	**77.06**	**0.2448**	**81.34**	**0.7706**	**0.0221**	**166 s**	**4 s**
		Hybrid RLBiL	97.89	97.89	97.94	0.7600	97.89	0.9794	0.0404	660 s	16 s
None	SWaT	RNN	99.64	99.64	99.64	0.1310	99.64	0.9964	0.0025	16666 s	32 s
		**LSTM**	**99.87**	**99.87**	**99.87**	**0.0498**	**99.87**	**0.9987**	**0.0023**	**12000 s**	**10 s**
		BiLSTM	99.87	99.87	99.87	0.0499	99.87	0.9987	0.0023	13200 s	33 s
		Hybrid RLBiL	99.66	99.66	99.66	0.1219	99.66	0.9966	0.0023	28000 s	71 s
	WADI	RNN	98.71	98.71	98.71	0.0778	98.86	0.9871	0.0208	36060 s	9 s
		**LSTM**	**98.86**	**98.86**	**98.86**	**0.0764**	**98.86**	**0.9886**	**0.0202**	**32000 s**	**8 s**
		BiLSTM	98.82	98.82	98.82	0.0699	98.82	0.9882	0.0207	40000 s	10 s
		Hybrid RLBiL	49.97	49.97	0.000	18.038	50.00	0.0000	0.0000	28000 s	28 s
	GHL	RNN	99.64	99.64	99.64	0.1310	99.63	0.9964	0.0025	960 s	29 s
		**LSTM**	**98.18**	**98.18**	**98.18**	**0.6562**	**98.02**	**0.9818**	**0.0000**	**600 s**	**9 s**
		BiLSTM	99.88	99.88	99.88	0.0499	99.87	0.9988	0.0023	920 s	33 s
		Hybrid RLBiL	49.97	49.97	0.0000	18.0381	50.00	0.0000	0.0000	1120 s	28 s

Bold values indicate a particular model performed better compared to others under consideration based on various evaluation metrics.

**Fig. 7:**
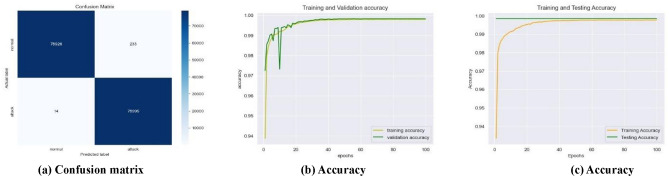
Best performance of the best-performing model at 0.001 learning rate for the SWaT dataset.

**Fig. 8:**
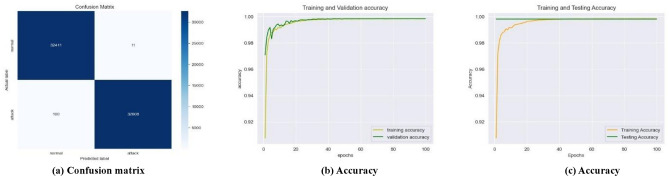
Best performance of the best-performing model at 0.1 learning rate for the WADI dataset.

**Fig. 9:**
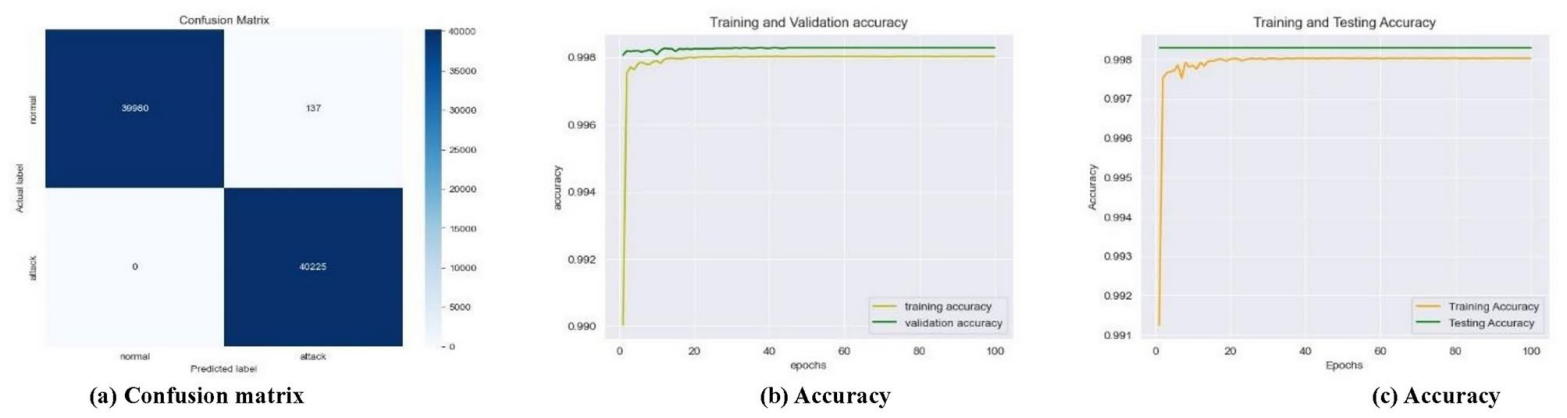
Best performance of the best-performing model at all the learning rates for the GHL dataset.

As the learning rate improves, and the number of epochs is incremented the number of false positives (FPs) and false negatives (FNs) decreases over time, and the testing error steadily drops, likewise, the testing accuracy exhibits an upward trend, the training time reduces over time. Nevertheless, the proposed framework demonstrated, a decrease in the number of FPs and FNs (Fig. 7a), an increase in training and validation accuracy (Fig. 7b), constant testing and increased training accuracy (Fig. 7c) for the SWaT dataset with the learning rate of 0.001, for the WADI dataset with the learning rate of 0.1 (Fig. 8a–c), and the GHL dataset with all the learning rates of 0.1–0.001 (Fig. 9a–c), respectively.

First, in terms of precision, recall, f1-score, log loss, ROC AUC score, and Z score the LSTM model in an interdisciplinary framework yielded better results when using ReLU to detect cyberattacks for the SWaT dataset, reaching up to 99.84% for the precision, 99.84% for the recall, 99.84% for the f1-score, 99.84% for the ROC AUC score, and − 0.9355 for the Z score (denotes a value that is below the average but still falls within the acceptable range). Second, for the WADI dataset, it reached 99.82% for the precision, 99.82% for the recall, 99.82% for the f1-score, 99.78% for the ROC AUC score, and − 0.4351 for the Z score (indicates an outcome that is somewhat below the average, but still within the range of what is considered usual). For the GHL dataset, it reached 99.82% for the precision, 99.83% for the recall, 92.73% for the f1-score, 99.82% for the ROC AUC score, and -∞ Z score (denotes a very negative data point that deviates significantly from the rest of the data) respectively as shown in Table 4.

Table 5 shows an evaluation of our framework on multiple datasets accompanied by multiple feature selection techniques. This is accomplished via a sigmoid layer, and the performance of the models is assessed using evaluation metrics such as accuracy, precision, recall, f1-score, and other parameters. Table 5 shows how the Deep LSTM model outperformed all the models proposed in the framework for all the datasets accompanied by multiple feature selection techniques, especially in MI and correlation-based techniques. In addition, training time in building the model and time taken in testing the model for the LSTM model shows a significant difference compared to the other models employed in the framework in all the cases.

**Fig. 10:**
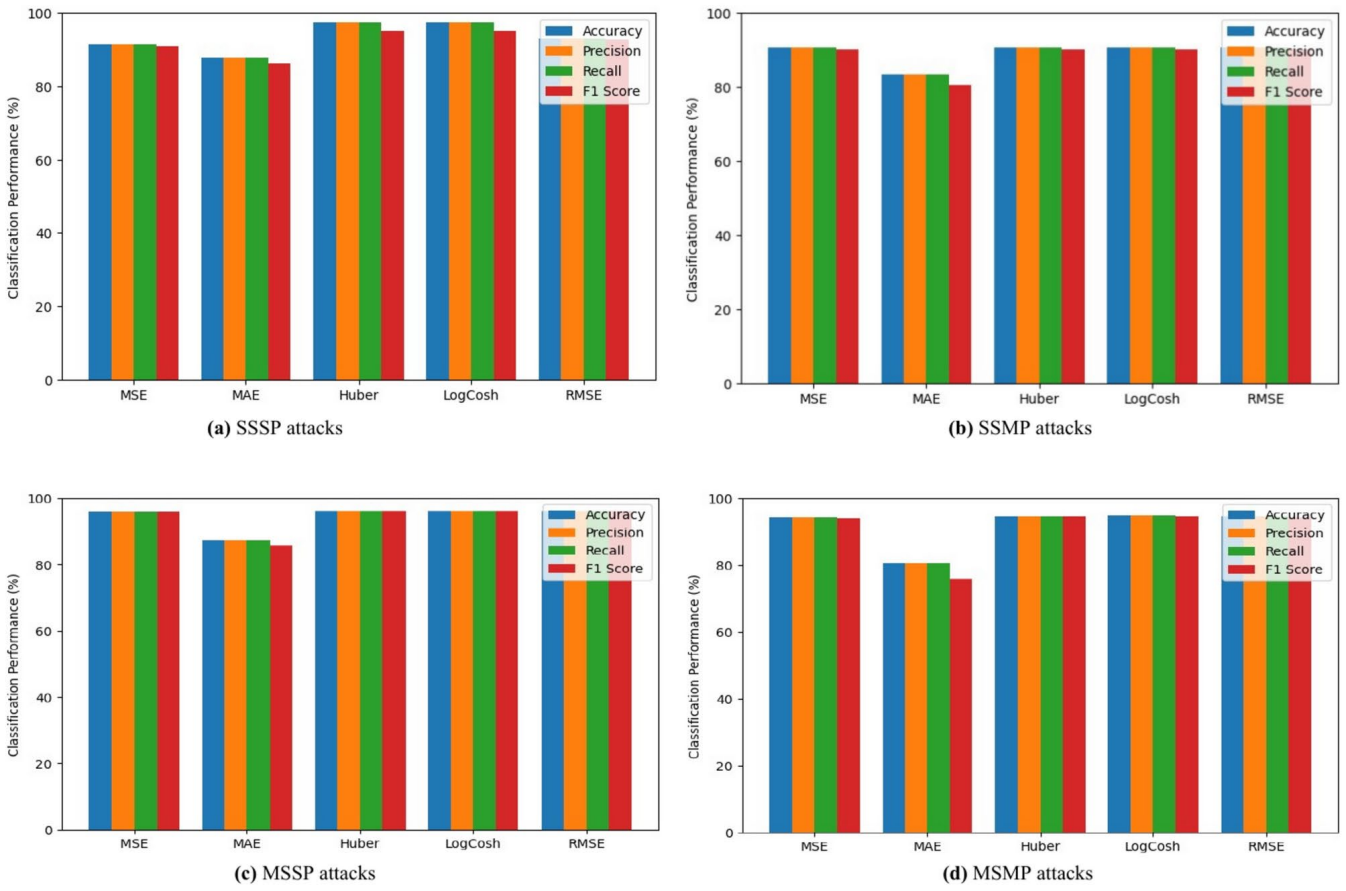
Performance evaluation of attack detection in SWaT dataset using multiple loss functions.

**Fig. 11:**
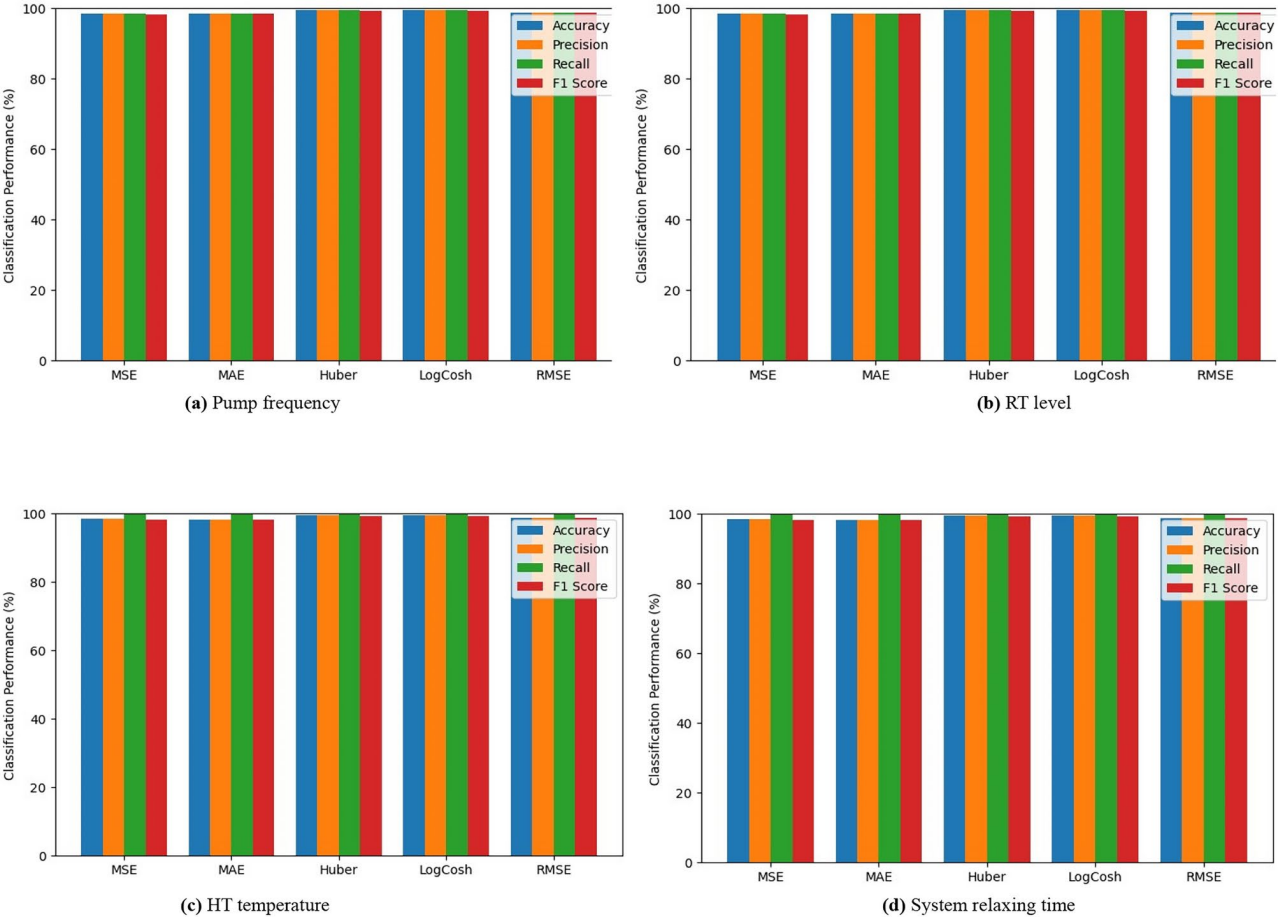
Performance evaluation of attack detection in the GHL dataset using multiple loss functions.

The SWaT dataset was utilized in a research study aimed at the detection of four distinct categories of attacks, as illustrated in Fig. 10. Based on the evaluation of accuracy, precision, recall, and F-score metrics, the detection of attacks exhibited the highest level of accuracy when employing the “RMSE” loss function. However, when it comes to the detection of attacks at the RT level and HT temperature in the GHL dataset Fig. 11b and c, this approach demonstrated a remarkable success in terms of superiority as per the recall metric. Nevertheless, the outcome proved a 100% success rate of the attack detection on the Pump frequency and on the System relaxing time Fig. 11a and d.

Our results suggest that, given ample computational resources, all the neural networks could attain a mean square error (MSE) within a narrow range of 0.0036 after a relatively small number of epochs. When we refer to sufficient computing resources, we specifically talk about the magnitude of the internal state for the Deep RNN, Deep LSTM, Deep Bi-LSTM, and Deep RLBiL models. We have noticed that this degree of inaccuracy may be attained by utilizing two or more layers. Increasing the number of layers does not have a substantial impact on improving the accuracy of the model. However, certain designs did show faster convergence compared to others.

**Fig. 12:**
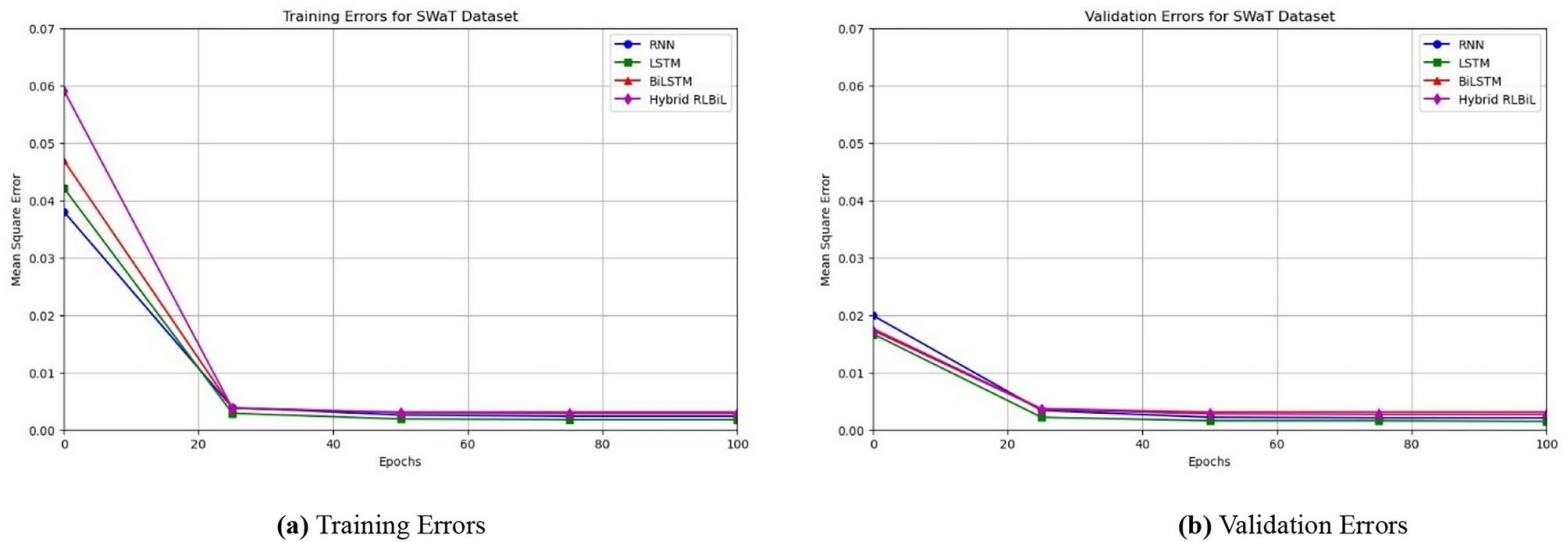
Training, and validation errors for the SWaT dataset.

**Fig. 13:**
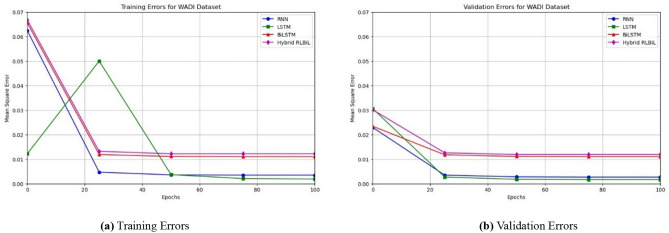
Training, and validation errors for the WADI dataset.

**Fig. 14:**
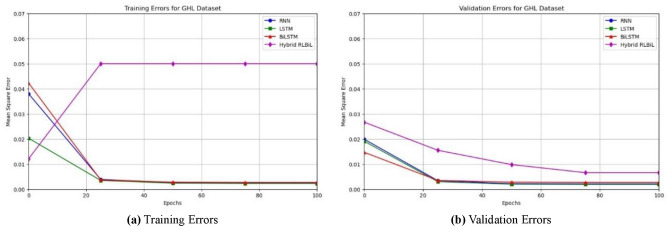
Training, and validation errors for the GHL dataset.

As seen in Figs. 12a, 13a and 14a, the LSTM and other configurations demonstrate rapid convergence and yield the lowest training error rate. However, the alternative configurations were not significantly worse and achieved comparable outcomes with more repetitions. As seen in Figs. 12b, 13b and 14b, the occurrence of validation mistakes gradually diminished, ultimately converging to a uniform range of errors. However, the LSTM model demonstrated superior convergence compared to the other models.

### Validation of the proposed framework on the related dataset

**Fig. 15:**
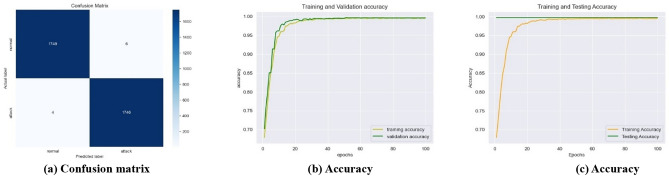
Best performance of the best-performing model at 0.1 learning rate for the BATADAL dataset.

**Fig. 16:**
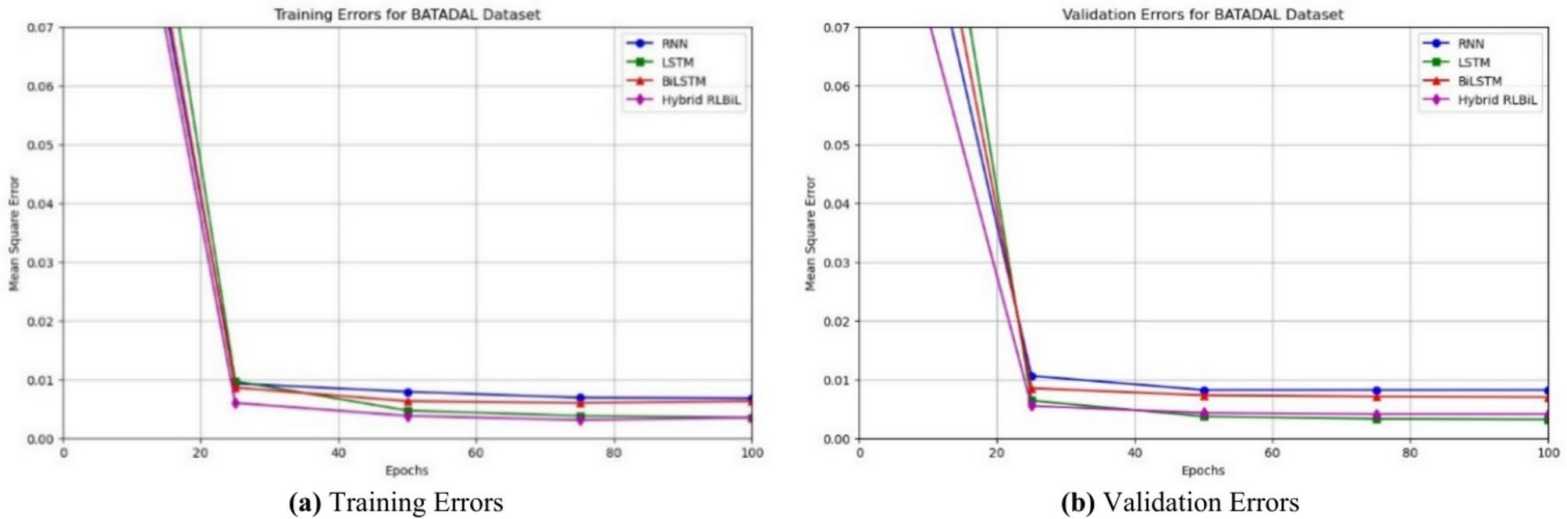
Training, and validation errors for the BATADAL dataset.

We validated our framework using the BATADAL dataset, representing a water distribution network. The network comprises seven storage tanks, eleven pumps, and five valves, making it a comprehensive simulation of real-world scenarios. The dataset was generated using epanetCPA^29,59^, a MATLAB toolbox that facilitates the injection of cyberattacks into the system. This toolbox also simulates the network’s response to these attacks, enabling detailed testing and analysis. The test dataset comprises 3505 records collected over 87 days of monitoring. This extensive data provides a robust basis for validating the effectiveness of our proposed framework. The findings for the BATADAL dataset are shown in Tables 6 and 7 respectively. Table 6 shows an evaluation of our framework on the BATADAL dataset accompanied by multiple feature selection techniques. Nevertheless, the proposed framework demonstrated, a decrease in the number of FPs and FNs (Fig. 15a), an increase in training and validation accuracy (Fig. 15b), constant testing and increased training accuracy (Fig. 15c) for the BATADAL dataset with the learning rate of 0.1. Figure 16a demonstrates that the LSTM setup displays swift convergence, attaining a minimal training error rate. Alternative arrangements, however somewhat less efficient, yielded comparable outcomes with increased repetitions. In Fig. 16b, validation errors progressively diminish over time, ultimately settling within a uniform error range. This illustrates the model’s capacity to enhance its performance across many settings.

**Table 6:** Parameter estimation.

Dataset	Learning rate	Training accuracy (%)	Testing accuracy (%)	Training precision (%)	Testing precision (%)	training recall	testing recall	Training F1-score	Testing F1-score	Z-score
BATADAL	**0.1**	**99.61**	**99.71**	**99.60**	**99.71**	**99.61**	**99.71**	**99.61**	**99.71**	− **0.4250**
	0.01	99.60	99.65	99.60	99.65	99.60	99.65	99.60	99.65	− 0.1225
	0.001	99.24	99.48	99.24	99.48	99.24	99.48	99.24	99.48	− 0.4197

Bold values indicate a particular model performed better compared to others under consideration based on various evaluation metrics.

**Table 7:** Validating framework on BATADAL dataset using multiple feature selection techniques.

Feature selection	DataSet	Algorithm	Precision	Recall	F1-score	Log loss	AUC score	TPR	FPR	Training time in building the model	Testing the model (s)
Mutual information	BATA-DAL	RNN	99.48	99.48	99.48	0.1851	99.48	0.9937	0.0039	300(s)	12 s
		**LSTM**	**99.71**	**99.71**	**99.71**	**0.1851**	**99.71**	**0.9977**	**0.0034**	**200(s)**	**16 s**
		BiLSTM	99.34	99.34	99.34	0.2365	99.34	0.9931	0.0062	400(s)	36 s
		Hybrid RLBiL	99.69	99.69	99.69	0.1131	99.69	0.9988	0.0051	900 s	80 s
Information gain	BATA-DAL	RNN	99.34	99.34	99.34	0.2365	99.34	0.9931	0.0062	300 s	20 s
		**LSTM**	**99.75**	**99.75**	**99.75**	**3.3319**	**99.75**	**0.8154**	**0.0005**	**100 s**	**10 s**
		BiLSTM	94.52	94.52	94.52	1.9744	94.52	0.8931	0.0028	200 s	16 s
		Hybrid RLBiL	94.01	94.01	94.01	2.1595	94.01	0.88	0.0000	400 s	30 s
Correlation coefficient	BATA-DAL	RNN	99.57	99.57	99.57	0.1543	99.57	0.9988	0.0074	200 s	16 s
		**LSTM**	**99.09**	**99.09**	**99.09**	**0.3291**	**99.09**	**0.9942**	**0.0125**	**200 s**	**16 s**
		BiLSTM	98.77	98.77	98.77	0.4422	98.77	0.992	0.0165	300 s	19 s
		Hybrid RLBiL	99.22	99.22	99.22	0.2811	99.22	0.9938	0.0094	300 s	22 s
None	BATA-DAL	RNN	99.14	99.14	99.14	0.3085	99.14	0.9908	0.0079	620 s	20 s
		**LSTM**	**99.29**	**99.29**	**99.29**	**0.2571**	**99.29**	**0.9914**	**0.0056**	**600 s**	**30 s**
		BiLSTM	99.37	99.37	99.37	0.2262	99.37	0.9942	0.0068	1200 s	55 s
		Hybrid RLBiL	50.84	50.84	50.84	18.0801	50.84	0.9996	0.9992	1400 s	82 s

Bold values indicate a particular model performed better compared to others under consideration based on various evaluation metrics.

### Comparison with other approaches

As illustrated in Tables 8, 9, and 10 our proposed solution achieved the highest precision among the evaluated methods for the specific metric under consideration. According to^16^, their findings indicate the second highest precision of 99.76% with the count of epochs being equal to 20 only, following our precision value of 99.84%. The authors^30^ attained the third-best precision of 98.40% moreover, our recall performance surpassed the mean of the recall scores reported in other studies. Eventually, our F1 score once again exceeded the mean value. Specifically^16^, achieved an F1-score of 99.76%, and a recall of 99.76% representing only the works that followed our findings.

**Table 8:** Result comparison of different methods using the SWaT dataset.

Method	Precision	Recall	F1-Score
1D CNN^19^	0.968	0.791	0.871
MLP^60^	0.967	0.696	0.812
CNN^60^	0.952	0.702	0.808
RNN^60^	0.936	0.692	0.796
LSTM^27^	–	–	0.817
TABOR^26^	0.823	0.862	0.788
DNN^25^	0.982	0.678	0.802
OCSVM^25^	0.925	0.699	0.796
MADICS^30^	**0.984**	0.750	0.851
DeepGCL^16^	**0.997**	**0.997**	**0.997**
DIF^6^	0.935	0.835	**0.882**
GAN^61^	0.700	0.954	0.810
Ours	**0.998**	**0.998**	**0.998**

Bold values indicate a particular model performed better compared to others under consideration based on various evaluation metrics.

**Table 9:** Result comparison of different methods using the WADI dataset.

Method	Precision	Recall	F1-score
PCA^61^	0.504	0.166	0.250
SVM^61^	0.512	0.512	0.510
KNN^61^	0.299	0.299	0.300
FP^61^	0.336	0.336	0.340
AE^61^	0.520	0.520	0.520
EGAN^61^	0.345	0.345	0.340
GAN^61^	0.538	0.749	0.520
DIF^6^	0.765	0.574	0.656
Ours	**0.997**	**0.997**	**0.997**

Bold values indicate a particular model performed better compared to others under consideration based on various evaluation metrics.

**Table 10:** Result comparison of different methods using the GHL dataset.

Method	Precision	Recall	F1-score
SPREAD^42^	–	–	–
LSTM^41^	0.976	0.788	0.872
DeepGCL^16^	0.976	0.956	**0.960**
Ours	**0.998**	**0.998**	0.997

Bold values indicate a particular model performed better compared to others under consideration based on various evaluation metrics.

The outcomes for detecting attacks on the SWaT system, as shown by the recall metric using a threshold value, are presented in Table 11 (the most optimal values are highlighted in bold). The present study compares the interdisciplinary framework approaches with established benchmark machine learning methods such as LSTM^27^, ID-CNN^19^, DeepGCL^16^, and MADICS^30^ models, as well as SVM^25^ and TABOR^26^.

**Table 11:** Recall comparison among multiple methods using SWaT Dataset.

Attack	Index #	Method										
		DNN^25^	RNN^60^	SVM^25^	TABOR^26^	1D-CNN^19^	DIF^6^	LSTM^27^	DeepGCL^16^	RNN	LSTM	BiLSTM
SSSP attacks	1	0	0	0	0.05	0.99	0.01	0	**0.997**	0.967	0.967	0.967
	2	0	0	0	0.93	**1.00**	0.29	1	0.968	0.909	0.955	0.965
	3	0	0	0	0	0.23	**1.00**	0	0.967	**1.000**	**1.000**	0.989
	4	0	0	0.04	0.33	0	0	0	**0.852**	0.551	0.551	0.551
	5	0.95	0.72	0.72	0.99	0.90	**1.00**	0	0.974	0.880	0.886	0.886
	6	0.91	0	0.89	0	1.00	**1.00**	0	0.996	0.934	0.934	0.934
	7	0.98	0.93	0.92	0.61	1.00	**1.00**	0.97	0.983	0.733	0.948	0.940
	8	0.98	1	0.43	0.99	1.00	**1.00**	0.95	0.936	0.918	0.918	0.918
	9	0.99	0.98	1	0.99	1.00	**1.00**	0.95	**1.000**	0.600	0.689	0.680
	10	0	0	0	0	0	0	**1**	0.413	0.910	0.910	0.910
	11	0	0	0	0	0	0.06	**1**	0.859	0.894	0.894	0.894
	12	0.60	0	0	0.24	0.24	0.55	0	0.888	0.980	**0.985**	0.980
	13	0	0	0	0.60	0.63	0.64	0	**1.000**	0.821	0.821	0.821
	14	0.97	0.12	0.13	0	0	0.45	0	0.978	0.865	0.846	0.864
	15	0	0.85	0.85	0.99	1.00	0.45	0	0.855	0.685	0.755	0.688
	16	0.98	0	0.02	0.08	0.91	0	0	**1.000**	0.997	1.000	0.997
	17	0.98	0.99	1	0.99	1	1	0.56	**1.000**	0.944	1.000	1.000
	18	0.71	0.88	0.88	0	**1**	0.82	0	0.945	0.944	0.971	0.944
	19	0.92	0	0	0	0.17	0.34	0	0.890	0.911	**0.961**	0.952
	20	0.29	0	0.01	0	0.02	**1**	0	0.975	0.757	0.757	0.7575
	21	0.99	0	0	0.99	**1.00**	0.17	0	0.965	0.882	0.936	0.882
	22	0	0	0	0.20	0.06	0	**1**	0.990	0.851	0.882	0.880
	23	0.03	0.94	0.94	1.00	1.00	**1.00**	0.94	0.995	0.893	0.933	0.991
SSMP attacks	24	0.87	0	0	0	0.06	**1.00**	0.53	0.989	0.986	0.989	0.989
	25	0.83	0	0	0.99	1.00	0	0.01	0.979	**1.000**	**1.000**	**1.000**
	26	0.78	0	0	0	0.30	**1.00**	1	0.990	0.957	0.957	0.860
	27	0.33	0	0.91	0	0.94	1.00	0.31	0.841	0.676	1.000	0.676
	28	0.84	0	0	0.88	0.89	0.43	**0.97**	0.920	0.933	**0.974**	**0.974**
	29	0	0	0	0.60	0.99	0	0	**0.999**	0.760	0.760	0.760
MSMP attacks	30	0	0	0	0.26	0	0.95	1	**1.000**	0.866	0.999	0.866
	31	0.81	0	0.12	0.89	0.88	0.93	0.34	**0.999**	0.894	0.894	0.894
	32	0.84	1	1	0.99	0.90	**1.00**	0.25	**1.000**	0.945	0.945	0.778
MSSP attacks	33	0.77	0.92	0.93	0.99	0.86	**1.00**	0.25	**1.000**	0.969	0.906	0.133
	34	0.84	0.94	0	0.40	0.91	**1.00**	0.67	**1.000**	0.604	1.000	0.604
	35	0.78	0.93	0.93	0.99	1.00	**1.00**	0	0.977	0.840	0.849	0.849
	36	0	0	0.36	0	0.64	0.63	0.89	0.972	0.966	**0.975**	0.966

Bold values indicate a particular model performed better compared to others under consideration based on various evaluation metrics.

An SSSP attack **(#1)** on opening MV-101 resulting in tank overflow was detected with 96.7% accuracy. An SSMP attack **(#26)** on LIT-401 by letting it have a value equal to 1000, keeping P-401 on resulted in tank overflow detected with a recall metric of 95.7%. An SSMP attack (#27) aimed at wasting the chemicals showed a 67.7% recall value (turning on P-201, turning on P-203, and turning on P-205). An MSMP attack **(#30)** aimed at damaging the RO was detected with a recall value of 86.6%. An anomalous behaviour caused by an MSSP attack **(#34)** on the P-302 constant power-up and LIT-401 overflow has been successfully detected, according to a recall metric of 100%. An MSMP attack **(#32)** aimed at tank 101 underflow, tank 301 overflow showed 94.5% of recall value (turning on P-101, MV-101 by letting LIT-101assume a constant value of 700 mm which causes P-102 to start itself due to a decrease in level in LIT301).

Additionally, the suggested framework underwent evaluation alongside other machine-learning approaches. A comparative analysis was conducted between 1D-CNN, MADICS, DIF, DeepGCL, DNN, and TABOR techniques on the SWaT dataset. The findings revealed that the LSTM model outperformed the other two models proposed in the framework. Notably, as highlighted in bold, the LSTM strategy produced the most favourable outcomes across most attack scenarios, as shown in the accompanying Table 11. It is noteworthy to acknowledge that assaults 29, 35 and 36 did not get recognition despite their lesser or greater performance compared to other approaches under consideration.

A total of 15 out of 15 WADI attacks were successfully identified, accounting for 100% of the attack scenarios, in contrast to [DIF]. A stealthy attack (**#3)** where an attacker wishes to drain an elevated reservoir 2_*LT_*002 was successfully detected with a recall score of 94.7%. An attack (**#9)** to turn on 1_*P_* 006 maliciously, resulting in a pipe burst, was detected with a recall metric of 94.3%. Attack (**#14)** aimed to pause chemical dosing to the raw water supplied to the primary grid tank, showing a recall value of 98.3%, as seen in Table 12.

**Table 12:** Recall comparison among multiple methods using WADI Dataset.

Attack	Method			
	DIF^6^	RNN	LSTM	BiLSTM
#1	0.000	0.856	**0.857**	**0.850**
#2	0.923	0.841	0.841	0.841
#3	0.923	0.944	**0.947**	**0.947**
#4	0.923	0.944	0.947	0.940
#5	**1.000**	0.961	0.961	0.961
#6	0.000	**0.750**	**0.750**	**0.750**
#7	**1.000**	0.904	0.944	0.940
#8	0.000	0.902	**0.909**	0.909
#9	0.911	0.943	**0.943**	0.941
#10	**1.000**	0.942	0.942	0.940
#11	**1.000**	0.903	0.909	0.903
#12	**1.000**	0.903	0.909	0.903
#13	**1.000**	0.944	0.956	0.946
#14	**1.000**	0.980	0.983	0.980
#15	0.856	0.888	**0.899**	0.888

Bold values indicate a particular model performed better compared to others under consideration based on various evaluation metrics.

The findings obtained from the experiments conducted on the GHL dataset demonstrated a significant level of accuracy for the suggested framework. The optimal values are shown in bold font. The analysis included examining four distinct categories of attacks that can potentially result in operational disruptions. Based on the recall metric, the event of an attack on the valve located inside the RT tank, resulting in the cessation of transferring heated fluid to the CT tank was detected with a recall rate of 100%. Similarly, a 100% recall rate was achieved in detecting an attack aimed at altering the system’s relaxing time value can be seen in Table 13.

**Table 13:** Recall comparison among multiple methods using GHL Dataset.

Attack	Method							
	LSTM^27^	1D-CNN^19^	GRU	DeepGCL^16^	DNN^25^	RNN	LSTM	Bi-LSTM
Pump frequency	0.813	0.511	0.998	0.998	0.993	**0.999**	**0.999**	**0.999**
HT temperature	0.927	**1.000**	0.345	**1.000**	0.987	0.997	0.997	0.997
RT Level	0.925	0.587	0.953	0.953	0.932	**1.000**	**1.000**	**1.000**
System relaxing time	0.602	1.000	0.979	0.999	0.967	**1.0000**	**1.000**	**1.000**

Bold values indicate a particular model performed better compared to others under consideration based on various evaluation metrics.

**Fig. 17:**
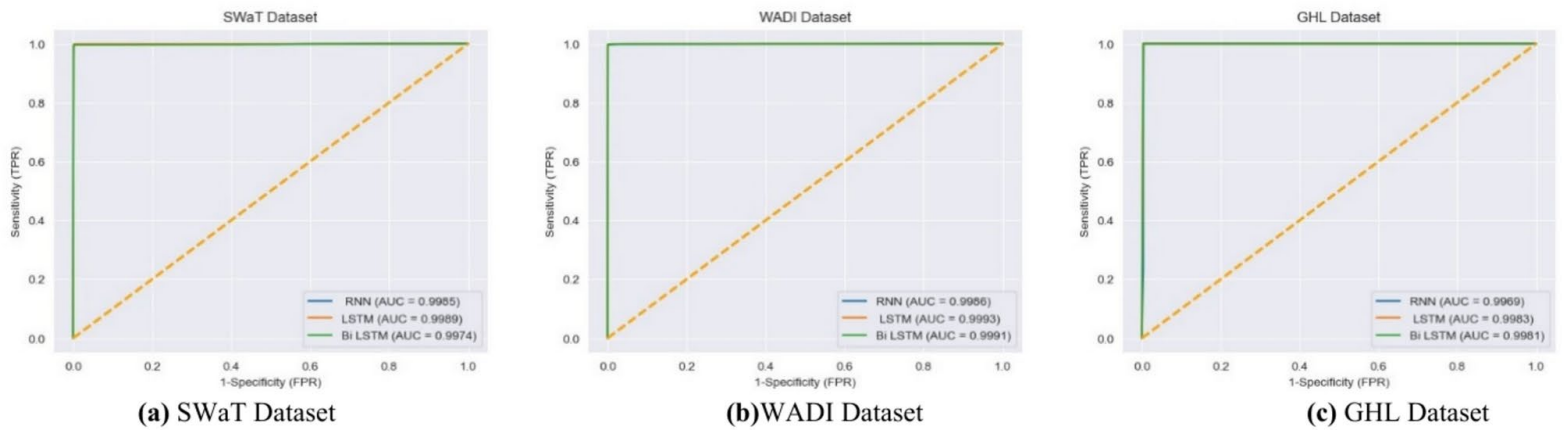
Multi-model ROC curves for the three datasets.

An attack on the change of maximum pump frequency and illegal altercation of max HT temperature was recognized by all the models of the framework equally. It exceeded the LSTM (81.3%) and 1D CNN (51.1%), whereas it descended in DeepGCL (99.8%) and GAN (99.8%). Likewise, an attack on illegal change of max HT temperature exceeded LSTM (92.7%), GRU (34.5%), descended in ID-CNN (100%), and DeepGCL (100%). The experimental findings indicate that the proposed framework for detecting attacks exhibits robustness and resilience compared to currently available machine learning techniques. The versatility of our anomaly detection system allows for its implementation across all types of Industrial Control Systems (ICS) since it relies on data obtained from sensors or actuators, irrespective of the specific process involved. The promotion of research is advocated to assess the viability of using the proposed attack detection approach in many additional industrial control system (ICS) applications, including power plants, transportation systems, and similar domains. Among the attention-enabled models employed in the framework, the Deep LSTM model yielded superior outcomes compared to the Deep RNN model, and the Deep Bi-LSTM model while simultaneously revealing shorter training durations and testing time. The given (Fig. 17a–c) i.e., the multi-model ROC curves for the three respective datasets reveal the exceptional performance of the LSTM model.

## Discussion

We examined several design factors to explain why the proposed deep learning-based NIDS framework surpasses current cyberattack detection approaches in ICS environments. The suggested framework has several advantages. It can differentiate between normal and malicious activities in ICS environments. This capacity is achieved by a training procedure that includes normal and malicious behaviours, enhancing the system’s ability to detect sophisticated cyber-attacks. Additionally, it provides an automated feature engineering approach that reduces the time and effort required while enhancing its efficacy in practical applications. The performance is contingent upon parameters modified exclusively during the training phase, allowing it to manage noisy and outlier data^62^.

**Fig. 18:**
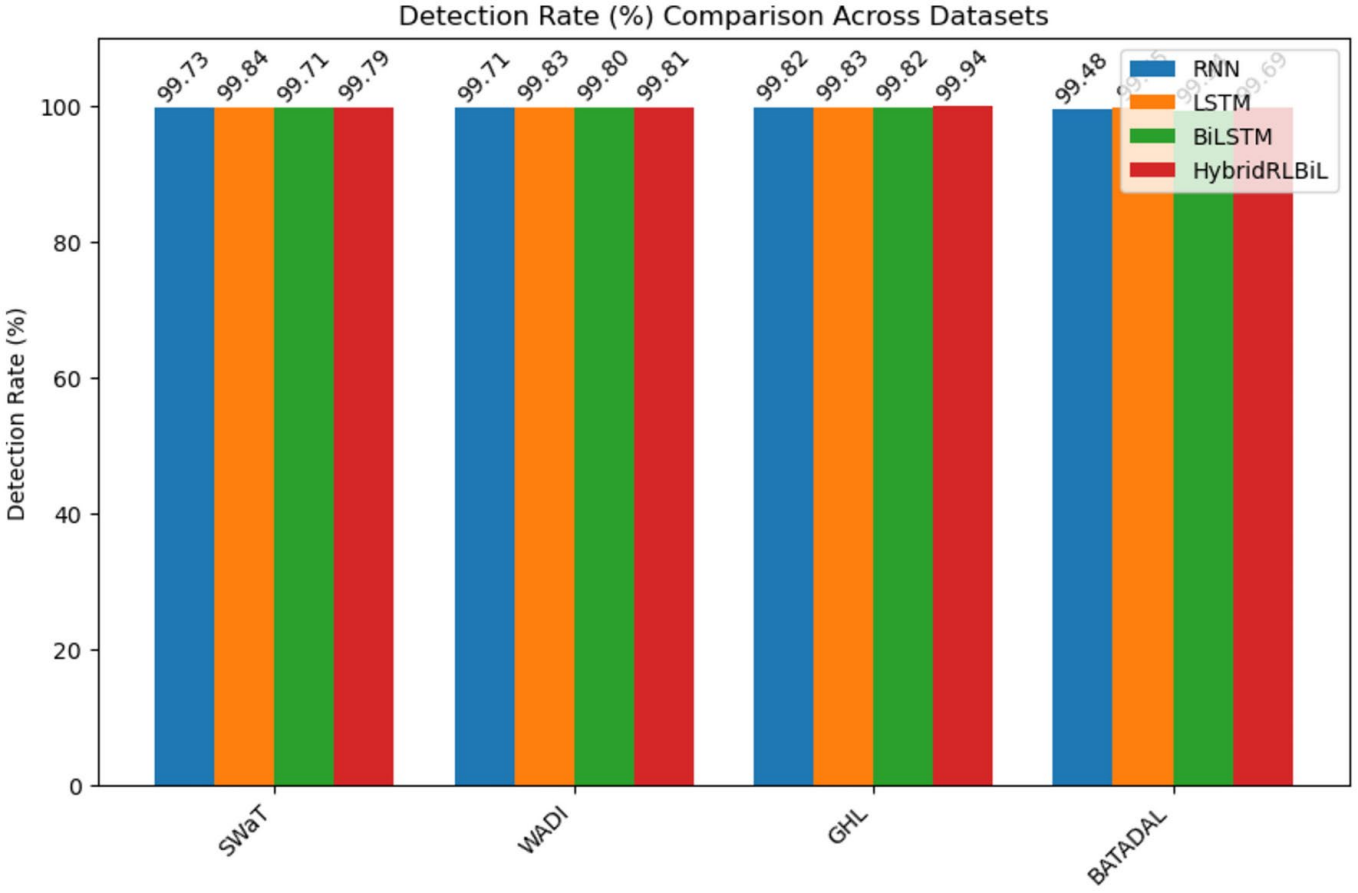
Detection Rate (%) across all the datasets employed in the proposed framework.

Based on the testing results, the proposed approach operates more swiftly than other ways of detecting abnormalities in real time and can efficiently differentiate between normal and attack data. For all the real-time datasets used in this experimental work the detection rate (%) attained by all three attention-driven deep learning models and the hybrid of these three deep learning models (see Fig. 18). From the deep learning models, attention-driven Deep LSTM model performed better except in the case of the GHL dataset where a hybrid model performed better compared to deep learning models but on account of higher training time (see Table 5).

A limitation of this method is the intricacy involved in parameter selection during the training phase. Nonetheless, this limitation is trivial, given the availability of advanced and high-performance hardware^62^. Furthermore, while the system is unable to handle non-numerical values beyond the original spectrum of feature values, this limitation may be alleviated with the application of feature transformation methods such as label encoding, one-hot encoding, and normalizing techniques during the preprocessing phase.

## Conclusions and future insights

Through this study, we examined the issue of detecting cyberattacks in Industrial Control Systems (ICSs) using deep learning-based network intrusion detection systems (NIDS). A specific interdisciplinary framework for cyberattack detection was suggested, which comprises feature selection and feature reduction with attention-driven neural networks: RNN, LSTM, and BiLSTM. It detects cyberattacks that result in malfunctioning in Industrial Control Systems (ICSs). Among the feature selection techniques, Mutual Information (MI) turned out to be better in selecting the optimum features for model training. The SparsePCA was employed to derive the higher-order features to expedite the convergence of each model suggested in the framework and enhance its resilience to outliers. The proposed framework was evaluated using the SWaT and WADI datasets from iTrust Lab, as well as the GHL dataset from Kaspersky Lab. These datasets contain information on the normal working of the system and instances of malfunctioning induced by cyberattacks. In testing the framework with different loss functions, root mean squared error (RMSE) was more effective in recognising cyberattacks and their vulnerability to outliers. Among the attention-driven deep learning models employed in the framework, the Deep LSTM model demonstrated better results in comparison to the Deep RNN model, Deep Bi-LSTM model, and the other models. This is evident from the results obtained and the shorter duration required for training and testing, as indicated in Table 4. The present benchmarks were evaluated with our proposed approach using precision, recall, and F1-score criteria, provided in Tables 8, 9, and 10, respectively. Moreover, Tables 11, 12, and 13 compare different state-of-the-art attack detection methods. Hence, our approach has demonstrated its superiority in detecting cyberattacks in Industrial Control Systems (ICSs).

Dataset balance is essential for augmenting learning efficacy and boosting cyberattack detection. The suggested framework, although limited to some specific datasets, exhibits flexibility in response to changing threats in Industrial Control Systems (ICS), guaranteeing strong performance. Future enhancements may concentrate on dynamic or real-time data balancing methodologies to effectively respond to evolving attack patterns. Second, applying the framework to other real-world datasets and complex attack scenarios will help validate its robustness and flexibility. Third, to offer an effective and strong security solution for ICS applications, big data, edge computing, federated learning, and blockchain-based security solutions must be adopted. These approaches will ensure promising, robust, and resilient security solutions in securing such ICS^35,63,64^ settings from advanced cyber-attacks.

## Data Availability

All the data sets listed in sub-section “Dataset description” can be accessed online for SWaT dataset:https://itrust.sutd.edu.sg/itrust-labs-home/itrust-labs_swat/, WADI dataset:https://itrust.sutd.edu.sg/itrust-labs-home/itrust-labs_wadi/, GHL dataset: https://ar5iv.labs.arxiv.org/html/1612.06676 and BATADAL dataset:https://itrust.sutd.edu.sg/itrust-labs_datasets/.

## Code availability

https://github.com/Qawsar2008854/Implementation-part.
